# Complement component C4 structural variation and quantitative traits contribute to sex-biased vulnerability in systemic sclerosis

**DOI:** 10.1038/s41525-022-00327-8

**Published:** 2022-10-05

**Authors:** Martin Kerick, Marialbert Acosta-Herrera, Carmen Pilar Simeón-Aznar, José Luis Callejas, Shervin Assassi, International SSc Group, P. Carreira, I. Castellvi, R. Ríos, R. García Portales, A. Fernández-Nebro, F. J. García-Hernández, M. A. Aguirre, B. Fernández-Gutiérrez, L. Rodríguez-Rodríguez, P. García de la Peña, E. Vicente, J. L. Andreu, M. Fernández de Castro, F. J. López-Longo, V. Fonollosa, A. Guillén, G. Espinosa, C. Tolosa, A. Pros, E. Beltrán, M. Rodríguez Carballeira, F. J. Narváez, M. Rubio Rivas, V. Ortiz-Santamaría, A. B. Madroñero, M. A. González-Gay, B. Díaz, L. Trapiella, M. V. Egurbide, P. Fanlo-Mateo, L. Saez-Comet, F. Díaz, J. A. Roman-Ivorra, J. J. Alegre Sancho, M. Freire, F. J. Blanco Garcia, N. Oreiro, T. Witte, A. Kreuter, G. Riemekasten, P. Airò, C. Magro, A. E. Voskuyl, M. C. Vonk, R. Hesselstrand, A. Nordin, C. Lunardi, A. Gabrielli, A. Hoffmann-Vold, J. H. W. Distler, L. Padyukov, B. P. C. Koeleman, Susanna M. Proudman, Mandana Nikpour, Australian Scleroderma Interest Group (ASIG), W. Stevens, J. Zochling, J. Sahhar, J. Roddy, P. Nash, K. Tymms, M. Rischmueller, S. Lester, PRECISESADS Clinical Consortium, Barbara Vigone, Jacques-Olivier Pers, Alain Saraux, Valérie Devauchelle-Pensec, Divi Cornec, Sandrine Jousse-Joulin, Bernard Lauwerys, Julie Ducreux, Anne-Lise Maudoux, Carlos Vasconcelos, Ana Tavares, Esmeralda Neves, Raquel Faria, Mariana Brandão, Ana Campar, António Marinho, Fátima Farinha, Isabel Almeida, Miguel Angel Gonzalez-Gay Mantecón, Ricardo Blanco Alonso, Alfonso Corrales Martínez, Ricard Cervera, Ignasi Rodríguez-Pintó, Gerard Espinosa, Rik Lories, Ellen De Langhe, Doreen Belz, Torsten Witte, Niklas Baerlecken, Georg Stummvoll, Michael Zauner, Michaela Lehner, Eduardo Collantes, Rafaela Ortega-Castro, Ma Angeles Aguirre-Zamorano, Alejandro Escudero-Contreras, Ma Carmen Castro-Villegas, María Concepción Fernández Roldán, Norberto Ortego, Enrique Raya, Inmaculada Jiménez Moleón, Enrique de Ramon, Isabel Díaz Quintero, Pier Luigi Meroni, Maria Gerosa, Tommaso Schioppo, Carolina Artusi, Carlo Chizzolini, Aleksandra Zuber, Donatienne Wynar, Laszló Kovács, Attila Balog, Magdolna Deák, Márta Bocskai, Sonja Dulic, Gabriella Kádár, Falk Hiepe, Velia Gerl, Silvia Thiel, Manuel Rodriguez Maresca, Antonio López-Berrio, Rocío Aguilar-Quesada, Héctor Navarro-Linares, Nicolas Hunzelmann, Gianluca Moroncini, Jeska K. de Vries-Bouwstra, Gisela Orozco, Anne Barton, Ariane L. Herrick, Chikashi Terao, Yannick Allanore, Carmen Fonseca, Marta Eugenia Alarcón-Riquelme, Timothy R. D. J. Radstake, Lorenzo Beretta, Christopher P. Denton, Maureen D. Mayes, Javier Martin

**Affiliations:** 1 Department of Cell Biology and Immunology, Institute of Parasitology and Biomedicine López-Neyra, CSIC, Granada, Spain; 2 Systemic Autoimmune Disease Unit, Hospital Clínico San Cecilio, Instituto de Investigación Biosanitaria Ibs. GRANADA, Granada, Spain; 3 Department of Internal Medicine, Valle de Hebrón Hospital, Barcelona, Spain; 4 Department of Internal Medicine, Hospital San Cecilio, Granada, Spain; 5 Department of Rheumatology, The University of Texas Health Science Center at Houston, Houston, TX USA; 6 Rheumatology Unit, Royal Adelaide Hospital and University of Adelaide, Adelaide, SA Australia; 7 The University of Melbourne at St. Vincent’s Hospital, Melbourne, VIC Australia; 8 Department of Dermatology, University of Cologne, Cologne, Germany; 9 Department of Clinical and Molecular Science, Università Politecnica delle Marche e Ospedali Riuniti, Ancona, Italy; 10 Department of Rheumatology, Leiden University Medical Center, Leiden, The Netherlands; 11 Center for Genetics and Genomics Versus Arthritis, Division of Musculoskeletal and Dermatological Sciences, School of Biological Sciences, Faculty of Biology, Medicine and Health, The University of Manchester, Manchester, UK; 12 NIHR Manchester Biomedical Research Center, Manchester University NHS Foundation Trust, Manchester, Greater Manchester UK; 13 Division of Musculoskeletal and Dermatological Sciences, The University of Manchester, Northern care Alliance NHS Foundation Trust, Manchester Academic Health Science Centre, Manchester, UK; 14 Laboratory for Statistical and Translational Genetics, RIKEN Center for Integrative Medical Sciences, Yokohama, Kanagawa Japan; 15 Department of Rheumatology A, Hospital Cochin, Paris, Île-de-France France; 16 Center for Rheumatology, Royal Free and University College Medical School, London, UK; 17 Center for Genomics and Oncological Research (GENYO), Pfizer-University of Granada-Andalusian Regional Government, Granada, Spain; 18 Department of Rheumatology and Clinical Immunology, University Medical Center Utrecht, Utrecht, The Netherlands; 19 Referral Center for Systemic Autoimmune Diseases, Fondazione IRCCS Ca’ Granda Ospedale Maggiore Policlinico di Milano, Milan, Italy; 20 Department of Rheumatology, 12 de Octubre University Hospital, Madrid, Spain; 21 Department of Rheumatology, Santa Creu i Sant Pau University Hospital, Barcelona, Spain; 22 Department of Rheumatology, Virgen de la Victoria Hospital, Málaga, Spain; 23 Department of Rheumatology, Carlos Haya Hospital, Málaga, Spain; 24 Department of Internal Medicine, Virgen del Rocío Hospital, Sevilla, Spain; 25 Department of Rheumatology, Reina Sofía/IMIBIC Hospital, Córdoba, Spain; 26 Department of Rheumatology, San Carlos Clinic Hospital, Madrid, Spain; 27 Department of Rheumatology, Madrid Norte Sanchinarro Hospital, Madrid, Spain; 28 Department of Rheumatology, La Princesa Hospital, Madrid, Spain; 29 Department of Rheumatology, Puerta de Hierro Hospital-Majadahonda, Madrid, Spain; 30 Department of Rheumatology, Gregorio Marañón University Hospital, Madrid, Spain; 31 Department of Internal Medicine, Clinic Hospital, Barcelona, Spain; 32 Department of Internal Medicine, Parc Tauli Hospital, Sabadell, Spain; 33 Department of Rheumatology, Hospital Del Mar, Barcelona, Spain; 34 Department of Internal Medicine, Hospital Universitari Mútua Terrasa, Barcelona, Spain; 35 Department of Rheumatology, Bellvitge University Hospital, Barcelona, Spain; 36 Department of Rheumatology, Granollers Hospital, Granollers, Spain; 37 Department of Internal Medicine, Hospital General San Jorge, Huesca, Spain; 38 Epidemiology, Genetics and Atherosclerosis Research Group on Systemic Inflammatory Diseases, DIVAL, University of Cantabria, Santander, Spain; 39 Department of Internal Medicine, Hospital Central de Asturias, Oviedo, Spain; 40 Department of Internal Medicine, Hospital Universitario Cruces, Barakaldo, Spain; 41 Department of Internal Medicine, Hospital Virgen del Camino, Pamplona, Spain; 42 Department of Internal Medicine, Hospital Universitario Miguel Servet, Zaragoza, Spain; 43 Department of Rheumatology, Hospital Universitario de Canarias, Tenerife, Spain; 44 Department of Rheumatology, Hospital Universitari i Politecnic La Fe, Valencia, Spain; 45 Department of Rheumatology, Hospital Universitari Doctor Peset, Valencia, Spain; 46 Department of Internal Medicine, Thrombosis and Vasculitis Unit, Complexo Hospitalario Universitario de Vigo, Vigo, Spain; 47 Department of Rheumatology, INIBIC-Hospital Universitario A Coruña, La Coruña, Spain; 48 Department of Clinical Immunology, Hannover Medical School, Hannover, Germany; 49 Department of Dermatology, Josefs-Hospital, Ruhr University Bochum, Bochum, Germany; 50 Clinic of Rheumatology, University of Lübeck, Lübeck, Germany; 51 Service of Rheumatology and Clinic Immunology Spedali Civili, Brescia, Italy; 52 Department of Rheumatology, VU University Medical Center, Amsterdam, The Netherlands; 53 Department of Rheumatology, Radboud University Nijmegen Medical Center, Nijmegen, Netherlands; 54 Department of Rheumatology, Lund University, Lund, Sweden; 55 Division of Rheumatology, Department of Medicine, Karolinska University Hospital, Karolinska Institute, Stockholm, Sweden; 56 Department of Medicine, Università degli Studi di Verona, Verona, Italy; 57 Istituto di Clinica Medica Generale, Ematologia ed Immunologia Clinica, Università Politecnica delle Marche, Ancona, Italy; 58 Department of Rheumatology, Oslo University Hospital, Oslo, Norway; 59 Department of Internal Medicine 3, Institute for Clinical Immunology, University of Erlangen-Nuremberg, Erlangen, Germany; 60 Department of Genetics, University Medical Center Utrecht, Utrecht, The Netherlands; 61 Menzies Research Institute Tasmania, University of Tasmania, Hobart, TAS Australia; 62 Department Rheumatology, Monash Medical Centre, Melbourne, VIC Australia; 63 Rheumatology, Royal Perth Hospital, Perth, WA Australia; 64 Research Unit, Sunshine Coast Rheumatology, Maroochydore, QLD Australia; 65 Canberra Rheumatology, Canberra, ACT Australia; 66 Department Rheumatology, The Queen Elizabeth Hospital, Woodville, SA Australia; 67 Centre Hospitalier Universitaire de Brest, Hospital de la Cavale Blanche, Brest, France; 68 Pôle de pathologies rhumatismales systémiques et inflammatoires, Institut de Recherche Expérimentale et Clinique, Université catholique de Louvain, Brussels, Belgium; 69 Centro Hospitalar do Porto, Porto, Portugal; 70 Katholieke Universiteit Leuven, Leuven, Belgium; 71 Medical University Vienna, Vienna, Austria; 72 Servicio Andaluz de Salud, Hospital Universitario Reina Sofía Córdoba, Córdoba, Spain; 73 Servicio Andaluz de Salud, Complejo hospitalario Universitario de Granada (Hospital Universitario San Cecilio), Granada, Spain; 74 Department of Medicine, University of Granada, Granada, Spain; 75 Servicio Andaluz de Salud, Complejo hospitalario Universitario de Granada (Hospital Virgen de las Nieves), Granada, Spain; 76 Servicio Andaluz de Salud, Hospital Regional Universitario de Málaga, Málaga, Spain; 77 Università degli studi di Milano, Milan, Italy; 78 Hospitaux Universitaires de Genève, Genève, Switzerland; 79 University of Szeged, Szeged, Hungary; 80 Charite, Berlin, Germany; 81 Andalusian Public Health System Biobank, Granada, Spain

**Keywords:** Structural variation, Genetic association study, Quantitative trait, Systemic sclerosis

## Abstract

Copy number (CN) polymorphisms of complement *C4* play distinct roles in many conditions, including immune-mediated diseases. We investigated the association of *C4* CN with systemic sclerosis (SSc) risk. Imputed total *C4, C4A*, *C4B*, and HERV-K CN were analyzed in 26,633 individuals and validated in an independent cohort. Our results showed that higher *C4* CN confers protection to SSc, and deviations from CN parity of *C4A* and *C4B* augmented risk. The protection contributed per copy of *C4A* and *C4B* differed by sex. Stronger protection was afforded by *C4A* in men and by *C4B* in women. *C4* CN correlated well with its gene expression and serum protein levels, and less C4 was detected for both in SSc patients. Conditioned analysis suggests that *C4* genetics strongly contributes to the SSc association within the major histocompatibility complex locus and highlights classical alleles and amino acid variants of *HLA-DRB1* and *HLA-DPB1* as *C4*-independent signals.

## Introduction

Systemic sclerosis (SSc) is a chronic immune-mediated inflammatory disease (IMID) more frequently observed in women (female/male ratio ~3.8–11.5) that affects the connective tissue and is associated with considerable morbidity and mortality^1–3^. The heterogeneous clinical manifestations of SSc are characterized by functional and structural vasculopathy, fibrosis of the skin and internal organs, in addition to inflammatory and immunological alterations like auto-antibody production^1^. The individual genetic background, together with environmental risk factors and epigenetics factors, play an important role in the pathogenesis of the disease^4,5^.

A recent genome-wide association study (GWAS) has identified new genes and pathways implicated in the development and progression of SSc^6^. Similar to other IMIDs, these genetic variations account for a limited portion of estimated heritability^7^, making clear that additional genetic variants remain to be found with the potential to bring novel insights into disease etiology and pathogenesis. In this sense, structural variants not captured by GWAS, such as copy number (CN) polymorphisms, which have been implicated in the etiology of several diseases^8,9^, could contribute substantially to the genetic risk of SSc. Several CN variants in immunological genes have been found to be associated with autoimmune diseases^10–13^, although technical limitations and the complexity of CN polymorphisms have reduced the impact of their analysis in understanding autoimmunity^14,15^.

The complement system plays an important role in innate immunity and forms a bridge to the adaptive immune response^16–18^. Functional abnormalities in the complement system have been widely described in rheumatic diseases, such as rheumatoid arthritis (RA) or systemic lupus erythematosus (SLE), and to a lesser extent in SSc^19^. Furthermore, genetic variability in several complement components may contribute to the development of inflammatory and autoimmune diseases^20,21^.

Complement component 4 (*C4*), encoded by two closely linked, highly polymorphic genes *C4A* and *C4B* within the major histocompatibility complex (MHC) class III region on chromosome 6, is an important protein in the classical and lectin complement activation pathways, which are major effectors for controlling microbial infections and for promoting clearance of apoptotic cells and soluble immune complexes^17,19^. *C4A* and *C4B* encode proteins with distinct affinities for their molecular targets^21–23^ and present variability in genomic copy number (CN)^24^ and length. The long form is determined by the presence of a 6.4-kb human endogenous retrovirus K (HERV-K) element in intron 9 of both genes. *C4* CN studies in autoimmunity have been mainly focused on SLE providing no definitive results^14,22,25^ in part due to the complexity of the genetic variation of *C4A* and *C4B* and the high linkage disequilibrium (LD) of the MHC locus, which contains the HLA genes, the strongest genetic associations with most autoimmune diseases including SLE, RA, and SSc^6,26,27^. A recent study, however, managed to attribute most of the genetic association of SLE and Sjögren’s syndrome (SjS) of the MHC locus to C4^28^. Of note, the same study made the complex genetic variation of *C4A* and *C4B* accessible through the imputation of SNP data using a large multi-ancestry panel of 2530 reference haplotypes^28,29^.

CN variations at *C4* genes have been implicated as a source of sexual dimorphism in two systemic autoimmune diseases, SLE and SjS^28^, diseases that have a higher prevalence in women than men. In light of the above, we set out to investigate the contribution of *C4* CN to SSc using data from the largest GWAS cohort of SSc, combined with genetic, RNA-sequencing, and C4 serum data from an independent cohort.

## Results

### Experimental design

To investigate the role of *C4* genetics in SSc, we analyzed two independent cohorts of European descent (see “Methods”, Supplementary Fig. 1 and Supplementary Table 1). We used the first cohort (genetic data, *N* = 26,633, 34% SSc, 10 countries) to determine the association of *C4* CN to SSc. The second cohort (genetic, whole blood expression, and C4 serum data, *N* = 857, 39% SSc, 9 countries) was analyzed to detect *C4* expression quantitative trait loci (eQTL) and for *C4* expression- and C4 protein level- modeling. Expression-model eQTLs from the second cohort that explained expression variance were subsequently used in the first cohort as co-factors to determine residual genetic association to SSc unrelated to *C4* genetics in the MHC region on chromosome 6. Finally, we utilized classical HLA alleles and amino acids (AAs) to model remnant *C4*-independent association in the MHC region.

### *C4* haplotype diversity and its correlation with classical HLA alleles

The complex genetic variation of *C4A* and *C4B,* which consists of many haplotypes with different numbers of *C4A* and *C4B* genes was recently made accessible for analysis in large cohorts^28^. We imputed 29 *C4* haplotypes independently in both cohorts. Haplotype frequencies were found to be similar in both cohorts and comparable to published results^28^ but varied substantially across countries (Supplementary Fig. 2 and Supplementary Table 2A). We determined nine *C4* haplotypes to have a moderate to strong correlation (*r*^*2*^ > 0.4) to at least one classical HLA allele (Supplementary Dataset 1). Correlation to classical HLA alleles associated to SSc on a genome-wide level was found to be small (*r*^*2*^ < 0.3) for most haplotypes with the exceptions of HLA-B*08:01 and HLA-C*07:01 which had a strong correlation (*r*^*2*^ = 0.82 and 0.67, respectively) with the BS haplotype and HLA-C*16:01 with AL-BS (H2111B) of 0.63 (Supplementary Dataset 1).

### The association between *C4* copy number variants and SSc is modified by sex and HERV-K

**Fig. 1:**
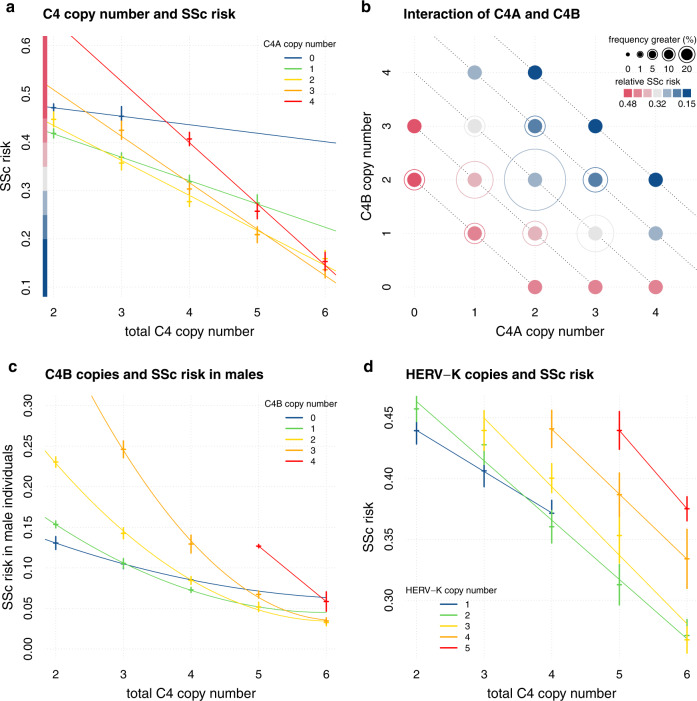
C4 and HERV-K copy numbers and Systemic Sclerosis risk. **a** depicts relative systemic sclerosis (SSc) risk vs total *C4* copy number stratified by C4A CN. The SSc risk score is calculated per individual as the sum of effect sizes (betas) multiplied with the design matrix. Betas of *C4A*, *C4B* and *C4A*:*C4B* were taken from the most complex model “d” (see “Methods”). Crosses are calculated as average relative risk per rounded *C4* CN + /− 2 standard deviations (*y* axis). Linear regression lines are colored by *C4A* CN and drawn to visualize the interaction effect of *C4A* and *C4B*. The *y* axis contains a color code to aid a comparison with (**b**). **b** depicts the relative SSc risk of combinations of *C4A* and *C4B* CNs. Relative risk is calculated as in (**a**). Outer circles are drawn according to population frequency ranges of each *C4A, C4B* combination and highlight more common combinations. Diagonal dotted lines help to identify combinations of equal total *C4* CN. **c** depicts relative SSc risk in male individuals vs total *C4* CN stratified by *C4B* CN. Relative risk is calculated like in (**a**) using effect sizes of *C4A*, *C4B*, *C4A:C4B*, *Sex:C4A*, and *Sex:C4B*. Crosses are calculated as average relative risk per rounded *C4* CN + /− 2 standard deviations (*y* axis). Cubic regression lines are colored by *C4B* CN and drawn to visualize the interaction effect of *C4A* and *C4B*. **d** depicts relative SSc risk vs total *C4* CN stratified by HERV-K CN. Relative risk is calculated like in (**a**) using effect sizes of *C4A*, *C4B*, *C4A:C4B*, *HERV-K:C4A*, and *HERV-K:C4B*. Crosses are calculated as average relative risk per rounded *C4* CN + /− 2 standard deviations (*y* axis). Linear regression lines are colored by HERV-K CN.

We found a higher *C4* CN to be protective for SSc (Fig. 1a and Table 1). Less than four copies of *C4* were found in 28.6% of SSc patients, and 2.8% had less than three copies. Interestingly, less than four copies of *C4* were found in 28% of female and 32% of male patients. In a simple additive model *C4A*, *C4B* and HERV-K CNs exhibited 1.8-fold variation in their relative risk of SSc (95% confidence interval (95% CI), [1.51–2.33]; *P* = 1.04 × 10^−16^). Logistic-regression analysis estimated a rather small difference between the protection afforded by each copy of *C4A* (odds ratio (OR) = 0.73; 95% CI = 0.69–0.78) or *C4B* (OR = 0.82; 95% CI = 0.77–0.87) (Table 1). We replicated our calculation in the second cohort and performed a meta-analysis, showing consistent results (Table 1).

**Table 1:** Logistic-regression analysis for total C4, C4A, C4B, and HERV-K copy numbers.

		1st cohort (*N* = 26,633)			2nd cohort (*N* = 857)			Meta-analysis		
Model	Model terms	Beta	s.e.	*P*	Beta	s.e.	*P*	Beta	s.e.	*P*
a: all	total C4	−0.23	0.03	6.3E-17	−0.20	0.13	0.12	−0.23	0.03	1.9E-17
	HERV-K	0.12	0.02	2.9E-10	0.16	0.10	0.10	0.12	0.02	7.9E-11
b: all	C4A	−0.31	0.03	3.7E-19	−0.36	0.18	0.04	−0.31	0.03	4.7E-20
	C4B	−0.20	0.03	7.0E-11	−0.14	0.14	0.33	−0.19	0.03	4.6E-11
	HERV-K	0.16	0.02	2.6E-13	0.23	0.11	0.04	0.16	0.02	3.4E-14
b: female	C4A	−0.27	0.04	6.5E-12	−0.81	0.33	0.01	−0.28	0.04	2.6E-12
	C4B	−0.22	0.03	1.1E-10	−0.10	0.27	0.70	−0.22	0.03	2.3E-13
	HERV-K	0.15	0.02	1.4E-10	0.41	0.19	0.03	0.15	0.02	1.5E-14
b: male	C4A	−0.49	0.08	1.7E-09	−1.19	0.48	0.01	−0.51	0.08	1.1E-10
	C4B	−0.09	0.07	2.3E-01	−0.73	0.43	0.09	−0.11	0.07	1.2E-01
	HERV-K	0.18	0.05	2.8E-04	0.66	0.28	0.02	0.19	0.05	7.5E-05
c: all	C4AShort	−0.29	0.18	1.0E-01	−0.43	0.32	0.18	−0.32	0.16	3.9E-02
	C4ALong	−0.16	0.02	2.0E-10	−0.11	0.18	0.55	−0.16	0.02	1.1E-15
	C4BShort	−0.20	0.03	1.4E-10	−0.12	0.28	0.67	−0.20	0.03	2.5E-11
	C4BLong	−0.04	0.03	1.8E-01	0.09	0.15	0.56	−0.04	0.03	2.3E-01

Depicted are beta values from the logistic-regression analysis of three different models (blocks of rows, see “Methods”). All models contained sex and five genetic principal components as co-variables. Logistic-regression analysis for the first cohort additionally contained cohort as co-variable. Model b was also calculated separately for females and males. Models contain copy numbers as calculated from the imputed *C4* alleles per individual as dosages.

The number of subjects in the first cohort permits the simple additive model to be expanded to a more complex one investigating the predictors that influence each other. We found evidence for three two-way interactions: an interaction of *C4A* with *C4B*, a sexual dimorphism of *C4A* and *C4B,* and an interaction of HERV-K CN with *C4A* and *C4B* (Supplementary Table 3). The full model *log(risk) ~ b*_*1*_ *Sex* + *b*_*2*_ *C4A* + *b*_*3*_ *C4B* + *b*_*4*_ *HERV-K* + *b*_*5*_ *Sex:C4A* + *b*_*6*_ *Sex:C4B* + *b*_*7*_ *HERV-K:C4A* + *b*_*8*_ *HERV-K:C4A* + *b*_*9*_ *C4A:C4B* predicted 7.7-fold variation in the relative risk of SSc. The following analyses have been derived from this complex model and its calculated coefficients.

The relationship between SSc risk and *C4A* and *C4B* gene CNs exhibited consistent, logical patterns across the 18 different CN combinations of *C4A* and *C4B* (Fig. 1a, b), which is based on an interaction between *C4A* and *C4B* CNs that was suggested by logistic-regression analysis (*b*_*c4a:c4b*_ = −0.14, *P* = 2.1 × 10^−5^). While higher total *C4* CN provide protection, strong deviations from the 1:1 ratio of *C4A* and *C4B* are of higher risk like e.g., four copies of *C4A* and zero copies of *C4B* (Fig. 1b).

We found evidence for a sexual dimorphism for *C4A* and *C4B* but not for HERV-K CN. Stratified analysis showed *C4A* to be more protective in men (*b*_*male*_ = −0.49, *P* = 1.7 × 10^−9^ vs *b*_*female*_ = −0.27, *P* = 6.5 × 10^−12^) while *C4B* showed statistical evidence to be protective only in women (*b*_*female*_ = −0.22, *P* = 1.1 × 10^−10^ vs *b*_*male*_ = −0.09, *P* = 0.23) (Table 1). Indeed, *C4B* CNs of two or higher seem to augment the risk for men (Fig. 1c). Logistic regression with interaction terms confirmed our results for the C4A:sex interaction, while the significance of the C4B:sex interaction was only suggestive (*b*_*male:c4a*_ = −0.17, *P* = 0.0034, *b*_*male:c4b*_ = 0.13, *P* = 0.065) (Supplementary Table 3). For total *C4* CN, we did not find a significant sex bias (*b*_*male:c4*_ = *−0.1, P* = 0.074). We calculated the statistical power for C4B association in males to be 0.41 and for the interaction of C4B:sex to be 0.33.

HERV-K copies generally augment the risk for SSc (Fig. 1d and Table 1). The protection afforded by *C4A* and *C4B* CNs are affected by HERV-K copies in a slightly different way. Protection associated with each *C4B* copy is affected more strongly by HERV-K (*b*_*HERV-K:C4B*_ = 0.085, *P* = 0.053) in comparison to *C4A* (*b*_*HERV-K:C4A*_ = 0.042, *P* = 0.03). A logistic-regression analysis with separate terms for the long and the short forms of *C4A* and *C4B* confirmed that the short forms confer more protection than the long forms (Table 1).

### *C4* copy number affects *C4* expression and C4 protein levels in whole blood

**Fig. 2:**
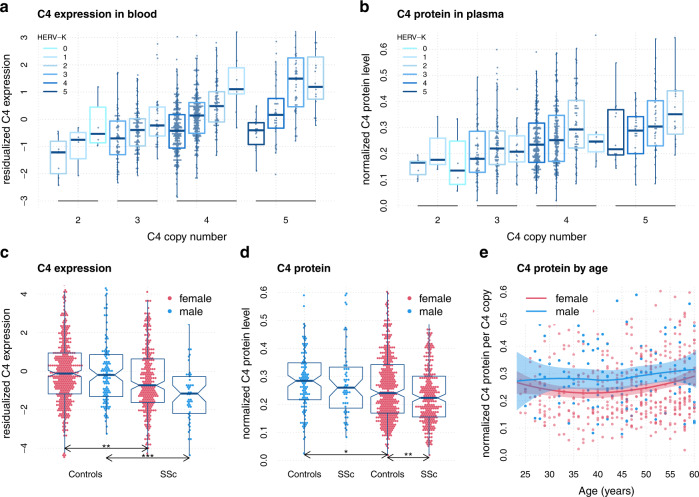
*C4* expression and C4 protein concentrations in whole blood. **a** depicts residualized total *C4* expression levels by total *C4* copy number (CN) stratified by HERV-K CN. *C4* expression is calculated as the sum of *C4A* and *C4B* expression as obtained by RNA-Sequencing. The residualized expression has been calculated by regressing out 20 (18) principal components for controls and cases, respectively. Data has been grouped by rounded *C4* and HERV-K CN dosage. **b** depicts normalized C4 protein levels in plasma by total *C4* CN stratified by HERV-K CN. C4 protein levels have been normalized across 10+ laboratory sites. **c** depicts residualized total *C4* expression levels (like in **a**) for SSc and controls, stratified by sex. Significant comparisons are highlighted by asterisk (*P < 0.05, **P < 0.01, ***P < 0.001). **d** depicts normalized C4 protein levels (like in **b**) for SSc and controls, stratified by sex. Significant comparisons are highlighted by asterisk (*P < 0.05, **P < 0.01, ***P< 0.001). **e** depicts normalized C4 protein levels in blood from 119 adult men (blue) and 447 adult women (red) as a function of age with locally estimated scatterplot smoothing (LOESS). Protein levels are normalized to the number of C4 gene copies in an individual’s genome. All boxplot are drawn with default settings in R 4.0.3: lines are defined as first, second and third quartile (Q1, Q2, Q3), whiskers depict the most extreme data points within Q1–1.5 interquartile range (IQR), and Q3 + 1.5 IQR. Boxplot notches are defined as 95% confidence interval of the median.

We investigated the effect of *C4* and HERV-K CN on whole blood expression levels of *C4* (*C4* = *C4A* + *C4B*, Fig. 2a). Specific analysis for *C4A* and *C4B* showed significant positive correlation for both *C4A* CN (*r*_*Pearson,C4A*_ = 0.15, *P* = 1.2 × 10^−5^) and *C4B* CN (*r*_*Pearson,C4B*_ = 0.34, *P* = 1.7 × 10^−24^) with *C4A* or *C4B* expression. HERV-K CN had a weakening effect on *C4* expression at all *C4* CN levels (Fig. 2a). *C4A or C4B* expression models performed better if they contained separate CNs for long (L) and short (S) versions of *C4* (AS, AL, BS, BL) instead of *C4A* plus *C4B* plus total HERV-K CNs. Interestingly, expression models were best if CNs of *C4B* were included in the model of *C4A* and vice versa. About 21% (27%) of expression variance of *C4A* (*C4B*) can be attributed to *C4A* and *C4B* CNs (Supplementary Table 4B, C).

Using *C4A* and *C4B* eQTLs alone, we were able to explain up to 42% (*C4B*: 38%) of expression variance with 19 (*C4B*: 15) SNPs (Supplementary Table 4B, C). This seems to be the upper bound of *C4* expression variance explained by *C4* genetics as *C4A* and *C4B* CN and eQTLs together could not explain more than 40% of expression variance albeit with only 12 (*C4B*: 13) additional SNPs (Supplementary Tables 4B, C). *C4* eQTLs seem to integrate *C4* and HERV-K CN information. Indeed, copy numbers of *C4A*_*Short*_, *C4A*_*Long*_, *C4B*_*Short*_, and *C4B*_*Long*_ can be predicted well (*r*^2^_C4AL_ = 0.5, *r*^2^_C4AS_ = 0.58, *r*^2^_C4BL_ = 0.54, *r*^2^_C4BS_ = 0.67, all *P* < 2.2 × 10^−16^) using *C4* eQTLs forward selected to explain *C4A* or *C4B* expression variance.

Blood serum concentrations of C4 protein were well correlated with *C4* CNs (*r*_*Pearson*_ = 0.25, *P* = 1.3 × 10^−12^, Fig. 2b). Regression analysis determined that *C4* and HERV-K CNs, sex, age, and SSc explained about 12% of C4 serum concentration variance (*P* = 1.4 × 10^−20^) with HERV-K copies again weakening C4 levels (*b*_*HERV-K*_ = −0.03, *P* = 9 × 10^−7^) (Fig. 2b and Supplementary Table 5). CN-corrected serum C4 levels determined men to have more C4 protein than women (*b*_*male*_ = 0.04, *P* = 4.6 × 10^−4^) independent of disease (Fig. 2e and Supplementary Table 5). Individuals with SSc had less CN-corrected C4 protein than healthy subjects independent of sex (*b*_*SSc*_ = −0.02, *P* = 0.012).

Despite that only about a third of SSc patients have less than four copies of *C4*, we found significant downregulation of *C4* expression (*p*_*female*_ = 0.001, *p*_*male*_ = 0.003) and C4 protein levels (*P* = 0.004) in SSc patients compared to healthy controls (Fig. 2c, d).

### *C4* genetics can explain a part of the SSc association to the MHC region

We performed conditional association analysis for genetic markers across the MHC genomic region. Conditioning on *C4A*, *C4B,* and HERV-K CN or on a risk score calculated using the complex *C4* CN interaction model derived above, showed an impact on residual association levels limited to the vicinity of the *C4* gene (Supplementary Fig. 3B, C). In addition, we calculated a *C4* risk score recently proposed for SLE and SjS based only on *C4A* and *C4B* CN (as: *risk* = (2.3)*C4A* CN + *C4B* CN)^28^. We applied the SLE/SjS score for conditional association analysis in the first dataset and found almost no effect (Supplementary Fig. 3A).

**Fig. 3:**
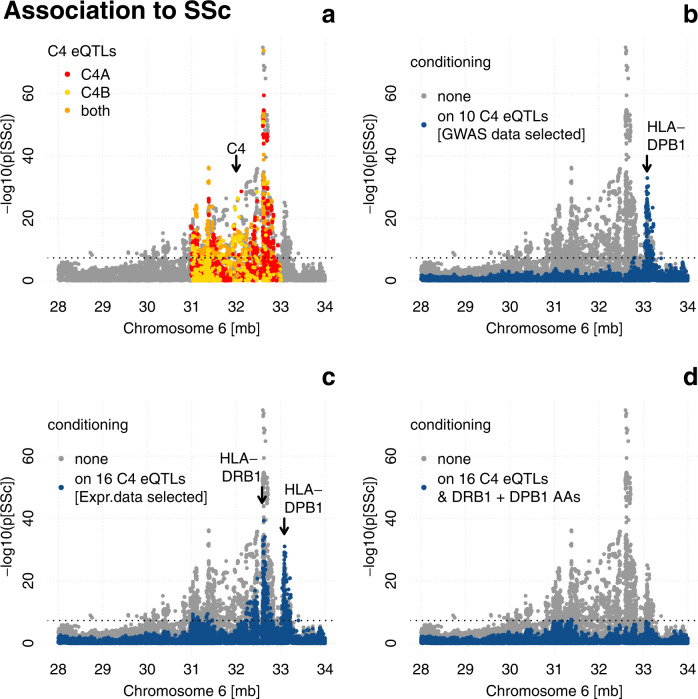
MHC region conditional association with systemic sclerosis. Association is calculated in the first dataset (*N* = 26,633) using logistic regression with cohort, genetic background (PC1-5), and sex as covariates and depicted as position (GRCh38) by significance (Manhattan plot) in gray if no additional covariates were used. The dotted line represents the genome-wide significance cutoff *P* = 5 × 10^−8^. **a** Manhattan plot with marked *C4* eQTLs obtained from the GTEx v8 database. **b** Manhattan plot with additional conditioning on ten independent *C4* eQTLs, obtained by forward selection in the first dataset, depicted in blue. The arrow marks the position of *HLA-DPB1*. **c** Manhattan plot with additional conditioning on 16 independent *C4* eQTLs (obtained by forward selection to explain expression variance in the second dataset (*N* = 857) depicted in blue. The arrows mark the positions of *HLA-DPB1* and *HLA-DRB1*. **d** Manhattan plot with additional conditioning on 16 independent *C4* eQTLs (obtained by forward selection to explain expression variance in the second dataset) and 8 independent amino acids of DRB1 and DPB1 (obtained by forward selection in the first dataset conditioning on 16 independent *C4* eQTLs) depicted in blue.

Given the strong association of *C4* CN with SSc and its rather local impact in conditional analysis, we focused on C4 eQTLs as potential modifiers of *C4* CN risk as both CN and eQTLs affect *C4* expression levels. Our analysis above suggests that the predictive power of *C4* CN and *C4* eQTLs on *C4* expression levels is at least partly redundant. In fact, *C4* eQTLs alone can explain more *C4* expression variance than *C4* CNs and *C4* eQTLs are significant predictors for copy numbers of *C4*. We obtained 10,680 eQTLs of *C4A* and *C4B* from the GTEx v8 database and found that 37% of these are associated to SSc with *p*_*GWAS*_ < 10^−5^ using the first dataset (Fig. 3a). This encouraged us to find independent *C4* eQTLs using forward selection to explain their contribution to the MHC association to SSc.

Conditioned association analysis for genetic markers across the MHC genomic region on ten independent *C4* eQTL SNPs rendered most association to SSc nonsignificant (*P* > 5 × 10^−8^), except for a peak around *HLA-DPB1*, the initial association of which was enhanced by conditioning (Fig. 3b). While conditioning on 13 independent *C4A* exclusive eQTL SNPs had a similar effect on the residual association profile, conditioning on 12 *C4B* exclusive eQTL SNPs had a smaller effect with residual association (*P* < 5 × 10^−8^) to SSc in three regions centered on *HLA-DPB1*, *HLA-DRB1,* and *HLA-B* (Supplementary Fig. 4B, C), which suggests a stronger contribution of C4A to SSc.

While the ten independent *C4* eQTL SNPs absorb as much SSc association as possible by design (forward selection), they might have been selected due to the extensive genetic linkage in the MHC region without being implicated in SSc pathogenesis. We therefore asked if SSc association with the MHC could be explained by *C4* eQTLs selected to explain *C4A* and *C4B* expression variance in the second dataset. Using expression-model eQTL SNPs, selected in the second cohort to explain expression variance, as co-factors in the first cohort to determine residual genetic association with SSc again rendered most MHC association with SSc nonsignificant (*P* > 5 × 10^−8^), except for the peaks shown in Fig. 3c centered on *HLA-DPB1* and *HLA-DRB1*.

### Remaining MHC signal after conditioning on *C4* genetics highlights *HLA-DRB1* and *HLA-DPB1*

Having attributed most SSc association within the MHC to *C4* genetics, we investigated which classical HLA alleles and HLA amino acids (AA) demonstrated *C4*-independent association to SSc. Conditioning on expression derived independent *C4* eQTLs results in residual significance (*P* < 5 × 10^−8^) for classical alleles and AAs of *HLA-DRB1*, *HLA-DPB1*, *HLA-DPA1* and *HLA-DQA1*, *HLA-DQB1*, and HLA-B (Supplementary Dataset 2A, B). Forward selection to derive independent residual signals marked HLA classical alleles for *HLA-DRB1* (*07:01, *11:03, *11:04, *13:01), *HLA-DPB1* (*13:01, *26:01, *40:01, *06:01), *HLA-DQA1* (*04:01), and HLA-DQB1*05:01.

We tested if classical alleles and AAs of *HLA-DRB1* and *HLA-DPB1* alone could explain residual *C4*-independent association. We found that 9 HLA classical alleles (*HLA-DRB1*: *11:04, *08:01, *07:01, *13:01, *11:03 and *HLA-DBP1*: *13:01, *26:01, *40:01, *06:01) together with 16 expression-model-derived independent *C4* eQTLs can explain almost all associations of SSc with the MHC region (Supplementary Fig. 5B). The same is true for AAs of HLA-DRB1 and HLA-DPB1, which can also explain residual *C4*-independent association with eight independent AAs (HLA-DRB1: 37SY, 58A, 74RL, 96E, and HLA-DPB1: 8, 76I, 91H, 96x) (Fig. 3d).

To complement our analysis, we repeated our search for residual *C4*-independent association to SSc, this time conditioning on the *C4* genetic signal which was not derived from the expression dataset but from the first dataset as ten independent *C4* eQTL signals as described. Repeating the forward selection of AAs or classical HLA alleles conditioning on the ten first dataset-derived independent *C4* eQTLs signals resulted in four independent AAs from *HLA-DPB1* (9F, 76I, 91H, 96x) or five independent alleles (*HLA-DRB1*: *11:04 and *HLA-DBP1*: *13:01, *26:01, *28:01, *30:01) (Supplementary Fig. 4D, E) supporting the role of *HLA-DRB1* and *HLA-DPB1* as a *C4*-independent association.

## Discussion

In this study, we found a strong association of low *C4* and high HERV-K CN with SSc in two independent SSc datasets and their meta-analysis, supporting the protective role of *C4* copies in IMIDs. *C4A* gene copies were slightly more protective than *C4B* as has been shown in SLE and SjS^28^ but our data suggest a complex interaction of *C4A* and *C4B* CNs that has to be evaluated in the context of HERV-K copies and sex. We found that in SSc, an equal number of *C4A* and *C4B* gene copies grants more protection than (strongly) imbalanced numbers which we found to be a risk for SSc (Fig. 1b). Our results might differ from recent observations in SLE and SjS where *C4A* and *C4B* copies have been described to act in an additive way, but the authors did not describe the interaction with HERV-K copies in detail^28^.

Our results showed a sexual dimorphism with respect to the protection afforded by *C4A* and *C4B*. While in female individuals, *C4A* copies grant slightly more protection than *C4B* copies, our data suggest that in male individuals only *C4A* confers protection while we did not observe a strong effect for *C4B*. In male individuals, *C4B* might therefore function like a null allele with respect to protection from SSc as higher CNs of *C4B* are associated to higher SSc risk (Fig. 1c). However, as the power of our study to detect significant C4B signals in males was limited, and the sex:C4B interaction was only of suggestive significance, we cannot rule out that C4B has a protective effect in males. While *C4* alleles have been described to act more strongly in men, no distinction was made between *C4A* and *C4B* activity in SLE or SjS in a recent study of similar size^28^. It has been described that activated C4A bonds preferably with protein antigens, such as immune complexes, while activated C4B reacts rapidly with carbohydrate antigens, such as bacterial cell walls^30^. This could partly explain the greater susceptibility to and severity of infections reported in men and the higher incidence of autoimmune diseases in women^31,32^. In addition, low *C4B* CNs have been associated to cardiovascular disease^33^ where the incidence in women is usually lower than in men^34^. Interestingly, in a recent paper studying the female-biased expression in human skin, several genes from the complement activation pathway were identified as a molecular signature and in genome-wide co-expression networks^35^. Sexual dimorphism has been extensively reported in vascular physiology and pathophysiology^36^, where women more commonly develop microvascular dysfunction, and in autoimmune-related interstitial lung diseases, where young women are most commonly affected^37^. All of these clinical manifestations, for which the role of *C4* is yet to be elucidated, are hallmarks of SSc.

Our data confirm that *C4* and HERV-K CNs are strong predictors of *C4* expression levels in blood and other tissues^29^. While the major site of *C4* expression is the liver^38^, it has been shown that whole blood can be used with some caution as a surrogate tissue for quantitative trait analysis^39–41^. In addition, local complement production by bone-marrow-derived monocytes and macrophages can restore humoral response in *c4* deficient mice^42^. Interestingly *C4A* and *C4B* expression models both profit strongly from the other gene’s CN as a predictor, which supports the genetic interaction between them suggested in this study. Furthermore, the distinction between the long and the short forms of *C4*: AS, AL, BS, and BL as expression predictors instead of *C4A*, *C4B*, and HERV-K CNs alone, greatly favors the accuracy of the expression model. This suggests that HERV-K acts specifically on the gene where it is located, to suppress its expression, which is supported by studies in brain and serum^22,29,43^. *C4A* and *C4B* CNs were able to explain about 20% of *C4A* and *C4B* expression variance, which is clearly lower than the ability of *C4* eQTLs, which could explain ~40%. Although we most likely over-fitted the expression data, SNPs seem to be the superior instrument in predicting *C4* expression as they can integrate CN as well as classical eQTL signals. This finding might help to bring C4 genetics to the clinic in the form of simple genetic tests. The interconnectedness of *C4* CN and eQTLs is further supported by *C4* eQTLs being able to predict *C4* CNs (AS, AL, BS, BL) with coefficients of determination of *r*^2^ > 0.5.

*C4* and HERV-K CNs are strong predictors of C4 protein levels in blood serum^43^. Interestingly, it was reported that men had on average 27% more C4 protein per *C4* CN than women and that this bias is stronger during reproductive years^28^. While we found the difference between men and women to be smaller; men had on average 17% more C4 protein per C4 CN than women; we think our dataset confirms this finding and its timeframe (Fig. 2f) reinforcing the role of *C4* in the differential susceptibility between men and women observed in SSc. The deficiency of *C4* may trigger an inappropriate clearance of apoptotic debris and stimulate chronic activation of myeloid cells. Also, it results in a defect to eliminate autoreactive B-cell clones, and a higher tendency to form self-reactive germinal centers^23^ and has been previously associated with more severe SLE with earlier disease onset^44^. We also observed that patients with SSc have lower C4 serum levels than unaffected individuals even after correcting for *C4* gene CN, suggesting that hypocomplementemia in SSc is not simply due to *C4* genetics but also reflects disease effects on background complement levels^45^.

*C4* expression was clearly down-regulated in SSc patients compared to healthy individuals, as were C4 protein levels, although to a lesser extent (Fig. 2c, d). This might be explained by the difficulty in standardizing C4 protein assays across 10+ laboratory sites but might also point to differential mRNA stability adding another layer of complexity yet to be analyzed. Indeed, while we observed clear disease-independent differences of C4 protein levels between men and women, there was no significant differential expression of *C4* between healthy men and women, which suggests post-translational effects to play a role. In this line it has been proposed that IFN-gamma increases the stability of *C3* and *C4* mRNA^46^ and a recent expression analysis in SSc detected a strong *IFN* signature in a subset of patients^47^.

More than a third of more than 10,000 *C4* eQTLs from the GTEx v8 database are associated with SSc (*p*_*GWAS*_ < 10^−5^) and *C4* eQTLs alone can be used to explain most of the association of SSc within the MHC region, further supporting their importance in SSc. Interestingly, *C4A*-specific eQTLs can explain more SSc association than *C4B*-specific eQTLs (Supplementary Fig. 4B, C), which supports a stronger role for *C4A* in SSc. While *C4* eQTLs could in principle be associated with SSc by the strong linkage structure present in the MHC, our data suggest that *C4* eQTLs, forward selected to explain expression variance in blood, can also explain most genetic association with the MHC. Both analyses raise the possibility that *C4* genetics is indeed the main signal on chromosome 6 for SSc, as has been suggested for SLE and SjS^28^, both rheumatic diseases that can co-occur with SSc.

*C4*-independent genetic association with SSc centers on two peaks (Fig. 3c), most of which can be explained by four AAs each of *HLA-DPB1* and *HLA-DRB1* (Fig. 3d). Interestingly, the AA positions for *HLA-DPB1* and *HLA-DRB*1 overlap and all 8 AAs positions can be associated with four of five binding pockets described for class II HLA molecules^48^ likely interfering with (auto-)antigen binding. In addition, three of the *HLA-DRB1* AA positions (37, 58, and 74) are close to sites (30, 60, and 74) which have been described to play a role in binding the consensus antigenic peptide of the topoisomerase I epitope, auto-antibodies to which define the ATA^+^ subgroup of SSc patients^49^. Furthermore, we found that the *C4*-independent genetic association with SSc can be explained by 10 independent classical HLA-Alleles instead of AAs, seven of which overlap with a model of nine independent HLA-Alleles recently described^50^, which supports the independence of *C4* and HLA associations with SSc.

Our study has some limitations. First, the number of samples in the second dataset is very low in terms of GWAS. While we were able to replicate the association of *C4A*, *C4B*, and HERV-K CNs, replication of the most complex model was out of reach and needs to be the subject of further study. Second, we did not stratify the patients by clinical or serological subgroups. While our results on HLA-DRB1 AAs, being associated with SSc independently of *C4* genetics, point towards anti-topoisomerase auto-antibodies and probably diffuse cutaneous SSc, the topic is too vast to explore in this study. Third, unfortunately, we could not distinguish *C4A* and *C4B* protein levels in serum, which would have been very useful, to further investigate the sexual dimorphism described. Fourth, the exact amino acid positions and classical alleles from the models calculated in our study might change in the future. New imputation reference panels might provide new associations that could influence the models as forward selection is sensitive to statistical fluctuations. Last, *C4* forms a genetic module termed RCCX with three genes: serine/threonine nuclear protein kinase *RP*, steroid 21-hydroxylase *CYP21*, and extracellular matrix protein tenascin *TNX*^51^. Although we only assessed *C4* CNs associated with SSc, we cannot discard the possibility that this module plays a role in disease susceptibility. Specifically, *TNX* is involved in the maintenance of collagen networks and tissue integrity^52^, as well as in TGFB activation and signaling, typical for fibrotic conditions such as SSc^53,54^.

Many rheumatic diseases, including SSc, could benefit from therapies directed toward the complement system. These are currently under active development and are not only focused on inhibitory mechanisms, but on activators or downstream activation fragments^23^. The inhibition of the complement pathway has proven challenging. Eculizumab, a C5 inhibitor, is a complement-targeting approved drug for a variety of vascular disorders and has recently been approved in kidney diseases^55^. Moreover, it has been studied in idiopathic inflammatory myopathies and SSc renal crisis, with promising results^56,57^. Our data suggest that *C4* genetics in SSc, by affecting expression and *C4* protein levels, plays an important role in mediating the genetic association in the MHC locus and might also be involved in the epidemiological sex bias of SSc. This highlights the contribution of the complement to the development of SSc and to autoimmune disorders in general, which could benefit from therapies directed towards the complement system. Our findings might help to bring C4 genetics to the clinic in the form of simple genetic tests.

## Methods

### Patients

All patients fulfilled the classification criteria of the 2013 American College of Rheumatology (ACR) or The European League Against Rheumatism (EULAR) or the criteria proposed by LeRoy and Medsger for early SSc^58,59^. CSIC’s Ethics Committee approved the study and written informed consent was obtained in accordance with the Declaration of Helsinki.

### Cohorts and datasets

We (re-)analyzed two independent cohorts of European descent:

First cohort: genome-wide genotyped data from 14 independent epidemiological cohorts comprising a total of 28,179 unrelated individuals (9846 SSc patients and 18,333 healthy subjects) from 10 European countries^6^. To identify ancestry outliers ~100,000 quality-filtered independent SNPs were selected from each case–control GWAS cohort. Principal component (PC) analysis was performed using PLINK v1.07. Samples showing >4 standard deviations from the cluster centroids of each cohort were considered outliers and removed from further analyses. The presence of relatives and/or duplicates was assessed by computing identity-by-descent (IBD) estimation using PLINK v1.07. An individual from each pair of relatives (Pi_Hat > 0.45) or duplicates (Pi_Hat > 0.99) was removed. After exclusion of non-European samples, we recalculated genetic PCs using the merge of all imputed datasets, selecting ~100,000 independent markers using PLINK v1.9. Missing data values due to the different platforms used for genotyping were corrected by PLINK v1.9 (parameter –correct_for_missingness). We obtained informative principal components as the visualization of the first two PCs can be interpreted as a “map” of the European continent (Supplementary Fig. 1). Second cohort: this cohort included genome-wide genotyped data, whole blood expression data and blood serum C4 protein concentrations from 333 SSc patients and 524 healthy individuals from 9 European countries^39^. This second cohort is a subset of a larger cohort of seven immune-mediated diseases plus controls described here^60^. Individuals were excluded on the basis of incorrect sex assignment, high missingness (>10%), non-European ancestry (<55% using Frappe^61^ and high relatedness (PLINK v1.9 Pi_Hat >0.5). In addition, population stratification was also analyzed by PC analysis selecting ~100,000 independent markers using PLINK v1.9. We obtained informative principal components as the visualization of the first two PCs can be interpreted as a “map” of the European continent (Supplementary Fig. 1).

Basic clinical epidemiological information by cohort can be found in Supplementary Table 1.

### Expression data

Whole blood expression data was obtained from alpha and beta globin depleted (globinCLEAR, Ambion) total RNA. Single end 50 bp stranded sequencing was performed on a HiSeq2500 Illumina within the PRECISESADS consortium^60^ and processed with bcl2fastq (Illumina), Cutadapt^62^, STAR v2.5.2 (2-pass default mapping to GRCh19^63^, and RSEM v1.2.31^64^ to obtain estimated counts per gene. Raw count data were normalized for quantitative trait analyzes^39^. Briefly: Three genetic principal components (PCs) were regressed out from VSN-normalized^65^ raw read count data. Potential non-genetic influences were regressed out for SSc and controls separately by 20 (SSc:18) PCs calculated from inter-sample expression correlation matrices.

### C4 protein data

Human complement C4 serum data was obtained from the PRECISESADS consortium^60^ from a turbidimetric immunoassay method according to the manufacturer’s recommendations (SPAPLUS analyzer)^66^. A corrective factor was calculated in order to normalize the data between the centers as described^60^.

### Imputation

#### SNPs

For both cohorts, we imputed SNPs from chromosome 6 using the TOPMed reference panel with default settings at https://imputation.biodatacatalyst.nhlbi.nih.gov/^67^. Stringent QC measures were applied to both cohort’s pre-imputation as follows: SNPs with call rates < 0.98; minor allele frequencies (MAFs) <0.01; and those that deviated from Hardy–Weinberg equilibrium (HWE; *P* < 0.001 in both case and control subjects) were filtered out from further analysis; samples with call rates <0.95 were removed. Relatives and/or duplicated samples were removed. Post-imputation quality control included filtered for imputation quality (*r*^*2*^ > 0.3), MAF > 0.05, and HWE, which ^6,39^ resulted in 9,068 SSc patients and 17,565 healthy individuals for *C4* haplotype imputation for the first cohort.

#### *C4* haplotypes

A set of 7021 SNPs TOPMed imputed SNPs were selected as they were (a) imputed in all individuals in both cohorts and (b) overlapped the *C4* CN reference panel. *C4* haplotype imputation was carried separately for both cohorts using the software imputec4^29^ and https://github.com/freeseek/imputec4 and the reference panel downloaded from the dbGaP study accession: phs001992.v1.p1^28^. Weighted imputation accuracy was calculated by multiplying *r*^*2*^_*Allele*_ by Allele frequency in Supplementary Table 2B.

#### *C4* copy numbers

Each *C4* haplotype carries a specific number of *C4* isotypes (*C4A*, *C4B)* and HERV-K elements (Supplementary Table 2C). We calculated total *C4*, *C4A*, *C4B*, and HERV-K CN dosages by multiplying the allele dosages of the structural haplotype by the number of copies of each *C4* isotype and HERV-K that the haplotype contains. For instance, the haplotype AL-BL contains one *C4A* gene and one *C4B* gene and two HERV-K copies. The numbers of short and long forms of *C4A* and *C4B* (AL, AS, BL, BS) per haplotype are self-evident for 17 of 29 imputed haplotypes. For the remaining, long and short forms were inferred by the consensus that ~95% of *C4A* is present in the long form^43,68–70^. The haplotype AL-BS for instance can be coded as 0.95 AL, 0.05 AS, 0.05 BL, and 0.95 BS. CNs per haplotype can be found in Supplementary Table 2C.

#### Classical HLA alleles and HLA amino acids (AA)

Data for the classical HLA alleles and AA variants were obtained from the first cohort by imputation using SNP2HLA^71^ and the reference panel from the Type 1 Diabetes Genetic Consortium^72^, described in ref. ^50^. After genotyping QC, all variants were imputed for each case–control dataset separately in the extended MHC region in chromosome 6. Imputed data were also filtered for 95% success call rate for alleles and amino acids, deviation from HWE considering a *P* value of <0.001 for SNPs in controls and 95% total call rate for individuals^50^.

### Pearson correlation of *C4* haplotypes and classical HLA Alleles

Was calculated among the C4 haplotype dosages and the allele dosages from the HLA imputation.

### *C4* copy number association analysis

Logistic-regression models from simple to complex were calculated (using the function glm in R 4.0.3) to assess the association of total *C4* dosage and its isotype dosages with the disease. We included cohort, five genome-wide principal components (PCs) and sex as covariates, assuming their effects were not collinear:

(a) SSc ~ *C4* + HERV-K + PC1-5 + cohort + sex

(b) SSc ~ *C4A* + *C4B* + HERV-K + PC1-5 + cohort + sex

(c) SSc ~ *C4A*_*short*_ + *C4A*_*long*_ + *C4B*_*short*_ + *C4B*_*long*_ + PC1-5 + cohort + sex

The number of subjects in our first cohort permits us to expand the simple additive model to a more complex one investigating the predictors that influence each other. We included three two-way interaction terms in the logistic-regression model:

(d) SSc ~ *C4A* + *C4B* + HERV-K + PC1-5 + cohort + sex + *C4A*:*C4B* + *C4A*: HERV-K + *C4B*: HERV-K + *C4A*:sex + *C4B*:sex

Meta-analysis was conducted with Metasoft^73^ using data from model a, b, and c from both datasets.

### Power calculation

Power calculations in CNV studies are problematic because effect sizes and models of the association are based on approximations that may be unrealistic^74^.

#### C4

Power calculations for C4B in males was carried out using the GAS Power Calculator [https://csg.sph.umich.edu/abecasis/gas_power_calculator/]. Here, we calculated the disease allele frequency as (sum(CN C4B < 2)/N) = 0.26 and the genotype relative risk as e^0.09^ = 1.09. Using an additive disease model and 1278 male cases and 6875 male controls, results in a power of 0.406 to detect an association with *P* < 0.05.

#### C4:sex interaction

We calculated the power to detect C4B:sex interactions using “powerGWASinteraction”^75^ in R 4.0.3 which can treat sex as environmental variable. We used: prevalence = 0.00034, pEnv = 0.306, betaC4B = −0.04, beta.sex = −1.35, beta.c4b:sex = 0.13, caseControlRatio = 0.34, ORgeneEnvironment = 1.03, alpha = 0.05 and alpha1 = 1. pGene probability was calculated as (sum(CN C4B > = 2)/*N*) = 0.73. This results in a power to detect a C4B:sex interaction with *P* values <0.05 of 0.33.

### Calculation of composite *C4* risk score for SSc

For each individual (i) a composite *C4* risk score can be calculated as the sum of betas “S_b,i_” from the (model-specific) effect sizes multiplied by the design matrix (of CN dosages, sex, interactions.. etc.) of the predictors. An individual relative risk score was then calculated as risk_i_ = e^Sb,i^/(1+e^Sb,i^).

To visualize the interaction effect of *C4A* and *C4B* CNs on relative risk, one multiplies the effect sizes (betas) of the most complex model “d” of *C4A*, *C4B*, and *C4A*:*C4B* with the design matrix and calculates the relative risk score as above. To visualize the effect of HERV-K on risk we calculated a composite score with model “d” betas and *C4A*, *C4B*, HERV-K, *C4A*:HERV-K, *C4B*:HERV-K, and *C4A*:*C4B*. To visualize the effect of *C4B* CN in males subjects, we used effect sizes from model “d” for sex, *C4A*, *C4B*, sex:*C4A*, sex:*C4B*, and *C4A*:*C4B*.

### Pearson correlation of *C4* CN and *C4* expression and C4 serum levels

Was calculated with *C4* CN dosages, the PC residualized expression data (see above) and the center corrected C4 protein serum concentrations. For visualization, CN dosages were rounded to integers.

### *C4* expression modeling

Total *C4* expression was calculated as the sum of *C4A* and *C4B* expression. We used the linear model function “lm” in R 4.0.3 to calculate the adjusted coefficient of determination (*r*^2^) for each model with *C4* CN, *C4* CN + *C4* eQTLs and *C4* eQTLs alone as predictors. Model evolution is noted in Supplementary Table 4A–C. To add *C4* eQTLs to the *C4* CN model as expression modifiers, we used forward selection. In a stepwise manner, we selected the SNP to add to the model which had the most significant *P* value conditioning on all predictors already in the model until no one more SNP was found with *P* < 0.01. In the same way, SNPs were selected for the eQTLs only model until no more SNPs were found with *P* < 0.01. To select SNPs in the expression (=second) dataset which were to be used for conditioned analysis of the MHC SNPs in the first dataset, forward selection was applied with SNPs which had *p*_*GWAS*_ < 10^−5^ until no more SNP was found with *P* < 0.01.

### Modeling of C4 copy numbers using eQTLs

We tested if the eQTLs found to explain *C4A* or *C4B* expression variance (see *C4* expression modeling) can predict copy number dosages of the long and short forms of *C4A* and *C4B*: AS, AL, BS and BL. We used the linear model function “lm” in R 4.0.3 to calculate the adjusted coefficient of determination (*r*^2^) for each model with either *C4A* eQTLs or *C4B* eQTLs as predictors.

### *C4* gene expression analysis in whole blood

Using raw count data, we included disease, blood cell composition, and effective library size (calculated by EdgeR in R 4.0.3) in the final model. While cell type-specific expression changes between SSc and controls were found significant at a nominal level for most cell types, the direction of expression change coincided for all cell types. We decided to report only whole blood expression changes controlling for blood cell composition.

### C4 protein blood serum analysis

We included disease, sex, age, AS, AL, BS, and BL in the final model. The significance for the difference between SSc and controls in men and women was calculated with both the Mann–Whitney test and a *t* test.

### Residual association of genetic variants across the MHC region to SSc

We performed conditional association analysis for genetic markers across the MHC genomic region. The first dataset was analyzed. In all models, we included cohort, five genome-wide PCs and sex as basic covariates. Association analysis of MHC region variants was conditioned on the basic covariates plus:

(1) nothing;

(2) a risk score: 2.3 × *C4A* CN + *C4B* CN as proposed;^28^

(3) covariates from model “b”: *C4A* CN + *C4B* CN + HERV-K CN;

(4) covariates from the most complex model “d” described above;

(5) *C4* (*C4A* or *C4B* or both) eQTLs from GTEx v8 (obtained by forward selection in the first dataset until no SNP had p_SNP_ < 10^−5^, see Supplementary Tables 4B, 6 and 4C, 6);

(6) *C4A* -specific eQTLs from GTEx v8 (obtained by forward selection in the first dataset until no SNP had *p*_*SNP*_ < 10^−5^). EQTLs were called *C4A*-specific if no *C4B* eQTL was reported in GTEx v8 with *P* < 0.01 for each SNP;

(7) C4B-specific eQTLs from GTEx v8 (obtained by forward selection in the first dataset until no SNP had *p*_*SNP*_ < 10^−5^);

(8) expression-model SNPs (with *p*_*GWAS*_ < 10^−5^) (obtained by forward selection in the second dataset as described above, see Supplementary Tables 4B, 7 and 4C, 7).

### Residual, *C4*-independent, the association of the MHC region with SSc

After accounting for the contribution of *C4* genetics with models “5” or “8” above, we sought to model residual, *C4*-independent, association of MHC SNPs with (a) forward selection of classical HLA alleles; (b) forward selection of classical HLA alleles of *HLA-DRB1* and *HLA-DPB1*; (c) forward selection of AAs of HLA genes; (d) forward selection of AAs of HLA-DRB1 and HLA-DPB1. Forward selection was carried out until no more HLA allele or AA was found with *P* < 5 × 10^−8^.

### Reporting summary

Further information on research design is available in the Nature Research Reporting Summary linked to this article.

## Supplementary information

Supplementary Files

Supplementary data 1

Supplementary data 2A

Supplementary data 2B

Reporting Summary

## Data Availability

Summary statistics of the SSc meta-GWAS are available through the NHGRI-EBI GWAS Catalog (https://www.ebi.ac.uk/gwas/downloads/summary-statistics): GCST009131. Data from the PRECISESADS consortium are hosted by ELIXIR Luxembourg https://elixir-luxembourg.org/ and are available upon request. The access procedure is described on the data landing page (10.17881/th9v-xt85). All other data are contained in the article file and its Supplementary Information.

## Code availability

All analysis has been performed with either the software described in “Methods” or within R 4.0.3. For logistic-regression analysis, the glm function of R was used.
