# Supplementary material for: Complement component C4 structural variation and quantitative traits contribute to sex-biased vulnerability in systemic sclerosis
Source: NPJ Genom Med. 2022 Oct 5;7:57. doi: 10.1038/s41525-022-00327-8 (PMC9534873; doi:10.1038/s41525-022-00327-8)
Supplement: Supplementary file 1 — Supplementary Files [file 41525_2022_327_MOESM1_ESM.pdf]

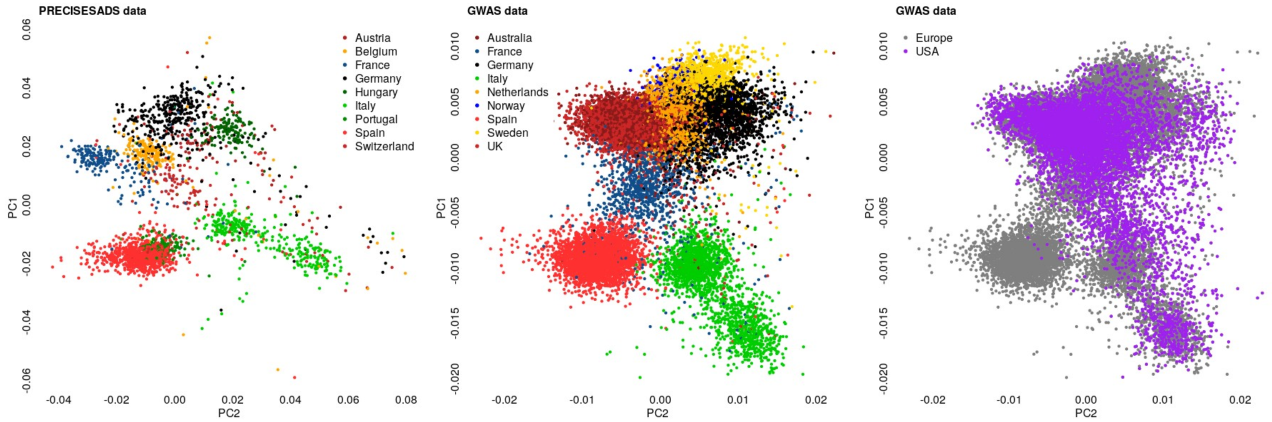

Supplementary Figure 1 The first two genetic principal components (PC) of both cohorts depict a "map of Europe".

After exclusion of non-european samples and quality control of imputed SNPs, we calculated genetic PCs using the merge of all imputed datasets, selecting ~ 100,000 independent markers using PLINK v1.9 (--indep-pairwise & --pca). Missing data values due to the different platforms in case of the first cohort (GWAS dataset, middle & right) used for genotyping were corrected by PLINK v1.9 (--correct for missingness). Depicted are colored by country of sampling **left:** the genetic PCs of the PRECISESADS cohort (N=2,534), a subset of which (controls and SSc samples, second cohort of this study, N=853) are analyzed in this study and **middle:** the genetic PCs of the first cohort of this study (N=26,633). On the **right:** are the genetic PCs of the first cohort (same as in the middle) of this study highlighting individuals obtained from the USA.

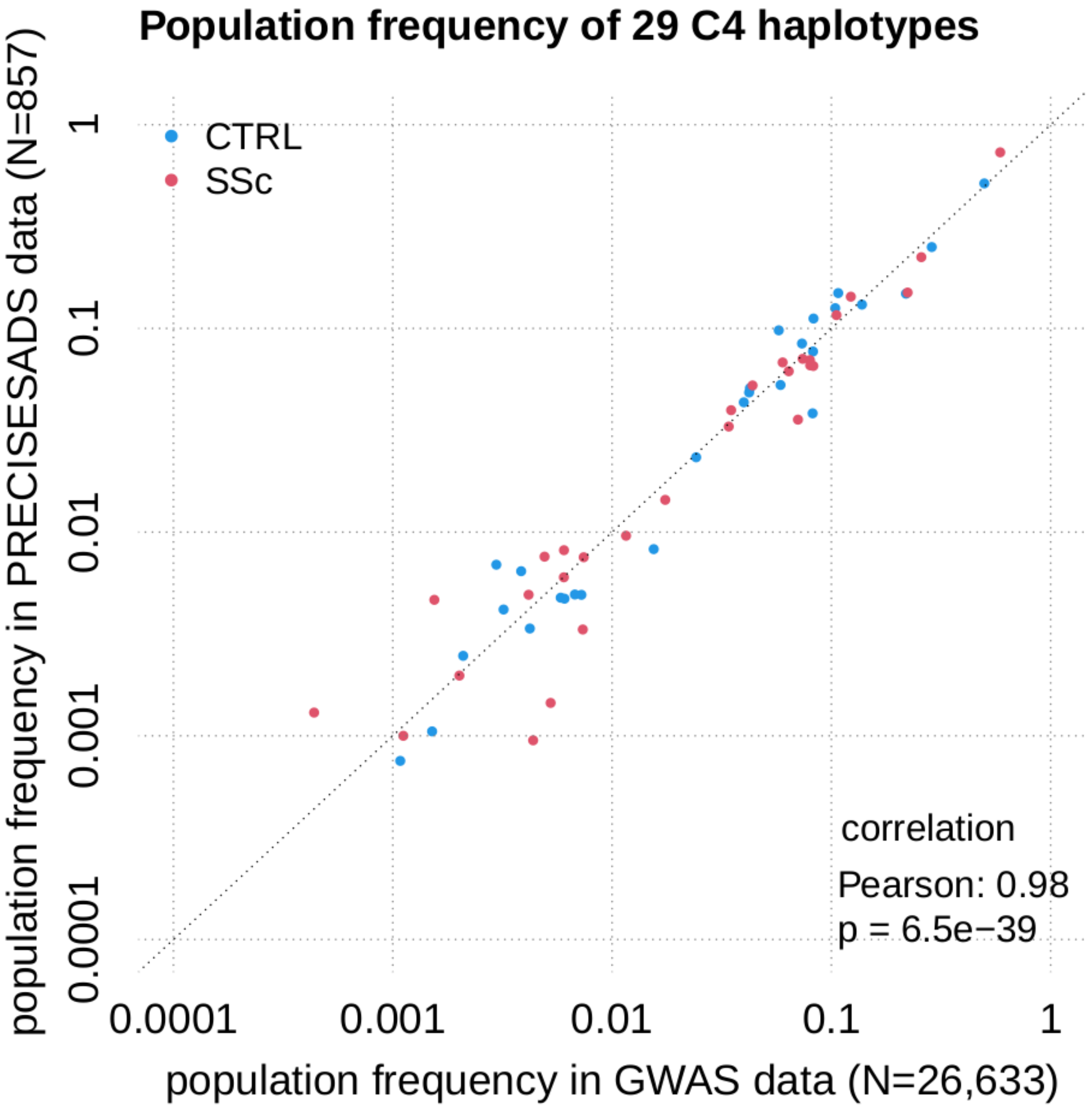

Supplementary Figure 2 Population frequency of 29 imputed *C4* haplotypes in two datasets

The x-axis depicts the haplotype frequency in the first dataset, the y-axis in the second dataset for SSc (red) and Control (blue) subjects. Haplotype frequencies have been scaled by log10 to ease visualization. The dashed line depicts Y = X.

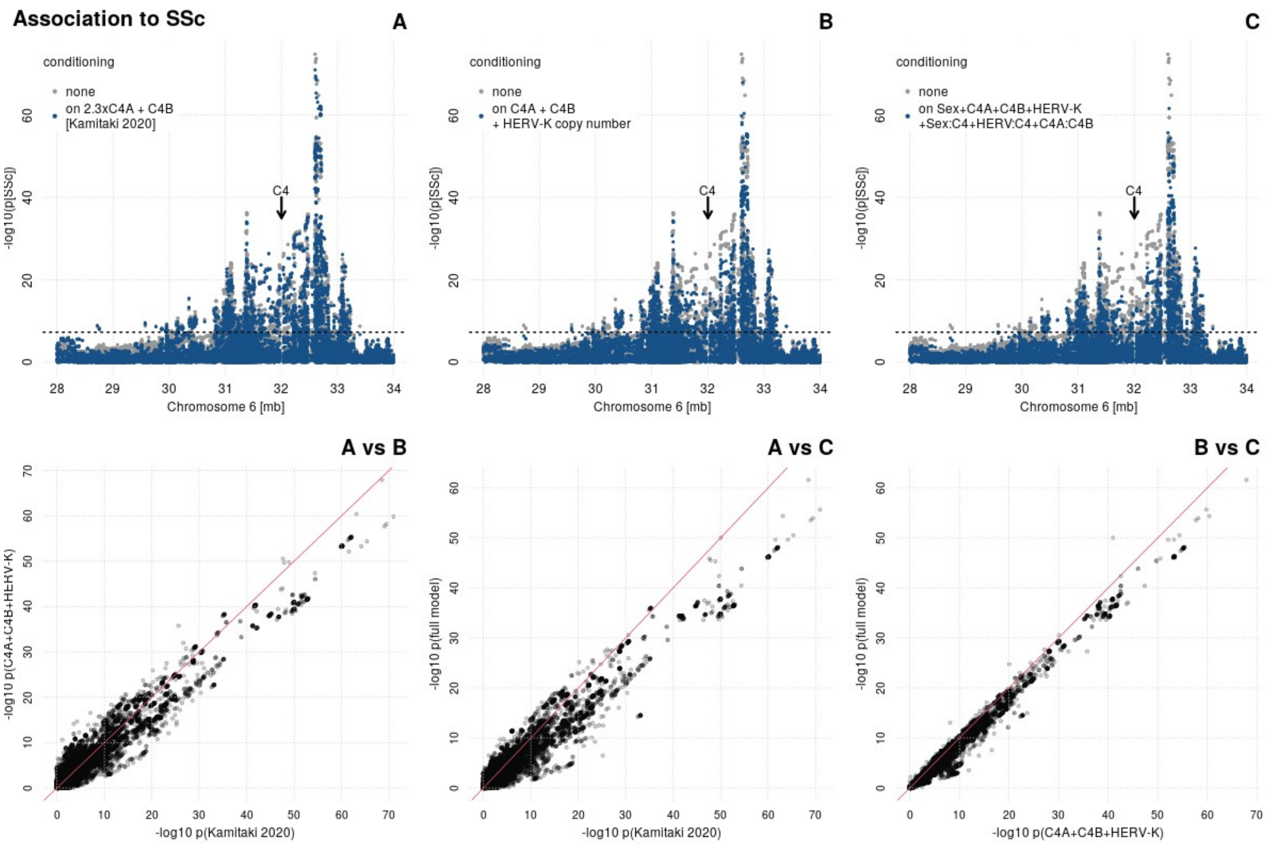

Supplementary Figure 3 MHC region conditional association with Systemic Sclerosis

Association is calculated using logistic regression in the first dataset (N=26,633) with cohort, genetic background and sex as covariates and depicted as position (GRCh38) by significance (Manhattan plot) in grey if no additional covariates were used. The arrow marks the position of *C4*. **A** Manhattan plot with additional conditioning (in blue) on 2.3x*C4A* CN + *C4B* CN as proposed by Kamitaki et al.[28] **B** Manhattan plot with additional conditioning (in blue) on *C4A* CN + *C4B* CN + HERV-K CN **C** Manhattan plot with additional conditioning (in blue) on the most complex model described: SSc ~ 1-5PC + cohort + sex + *C4A* + *C4B* + HERV-K + *Sex:C4A* + *Sex:C4B* + *HERV-K:C4A* + *HERV-K:C4B* + *C4A:C4B*.

The second row depicts comparisons of the conditional p-values against each other.

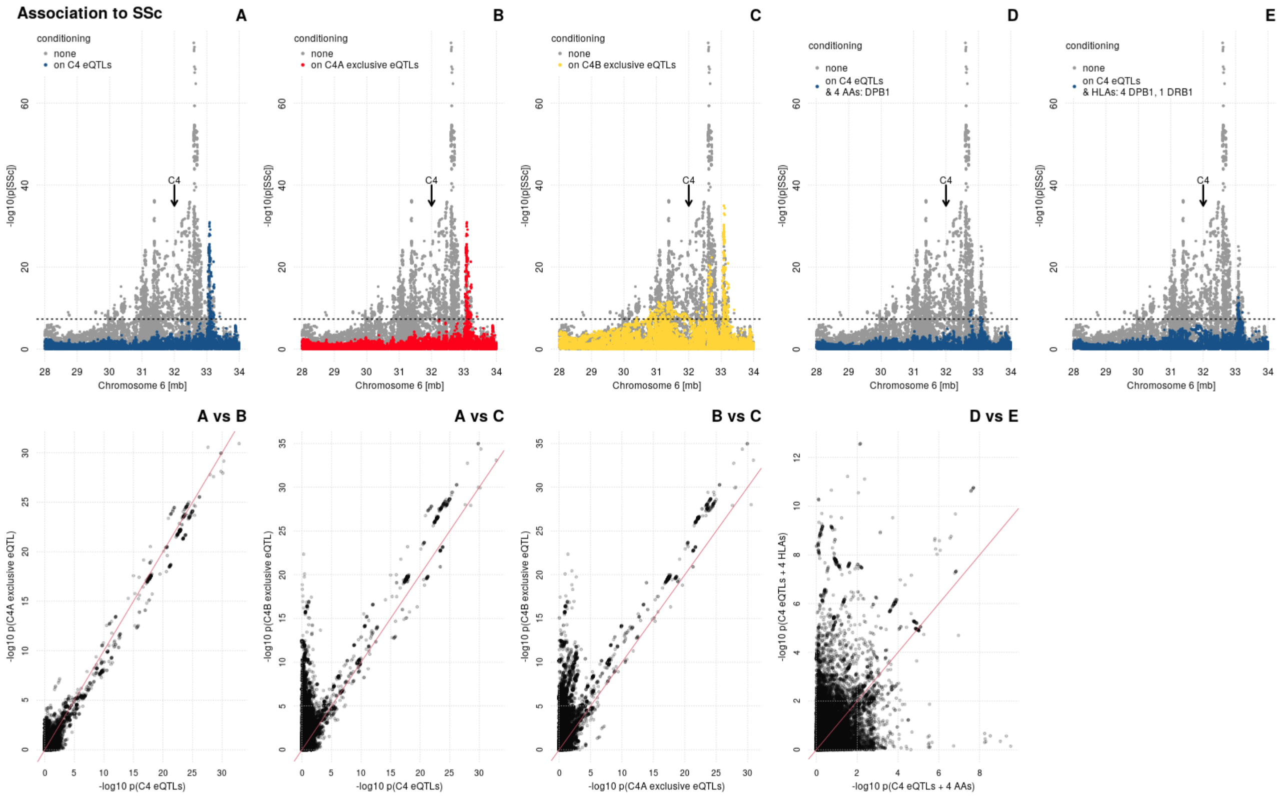

Supplementary Figure 4 MHC region conditional association with Systemic Sclerosis

Association is calculated using logistic regression in the first dataset (N=26,633) with cohort, genetic background and sex as covariates and depicted as position (GRCh38) by significance (Manhattan plot) in grey if no additional covariates were used. The arrow marks the position of *C4*. **A** Manhattan plot with additional conditioning on 10 independent *C4* eQTLs, obtained by forward selection in the first dataset, depicted in blue. **B** Manhattan plot with additional conditioning on 13 independent *C4A* exclusive eQTLs, obtained by forward selection in the first dataset, depicted in red. **C** Manhattan plot with additional conditioning on 12 independent *C4B* exclusive eQTLs, obtained by forward selection in the first dataset, depicted in orange. **D** Manhattan plot with additional conditioning on 10 independent *C4* eQTLs (obtained by forward selection in the first dataset) plus 4 AAs of HLA-DPB1 (obtained by forward selection in the first dataset conditioning on 10 independent *C4* eQTLs) depicted in blue. **E** Manhattan plot with additional conditioning on 10 independent *C4* eQTLs (obtained by forward selection in the first dataset) plus 4 classical alleles of *HLA-DPB1* and 1 classical allele of *HLA-DRB1* (obtained by forward selection in the first dataset conditioning on 10 independent *C4* eQTLs) depicted in blue.

The second row depicts comparisons of the conditional p-values against each other.

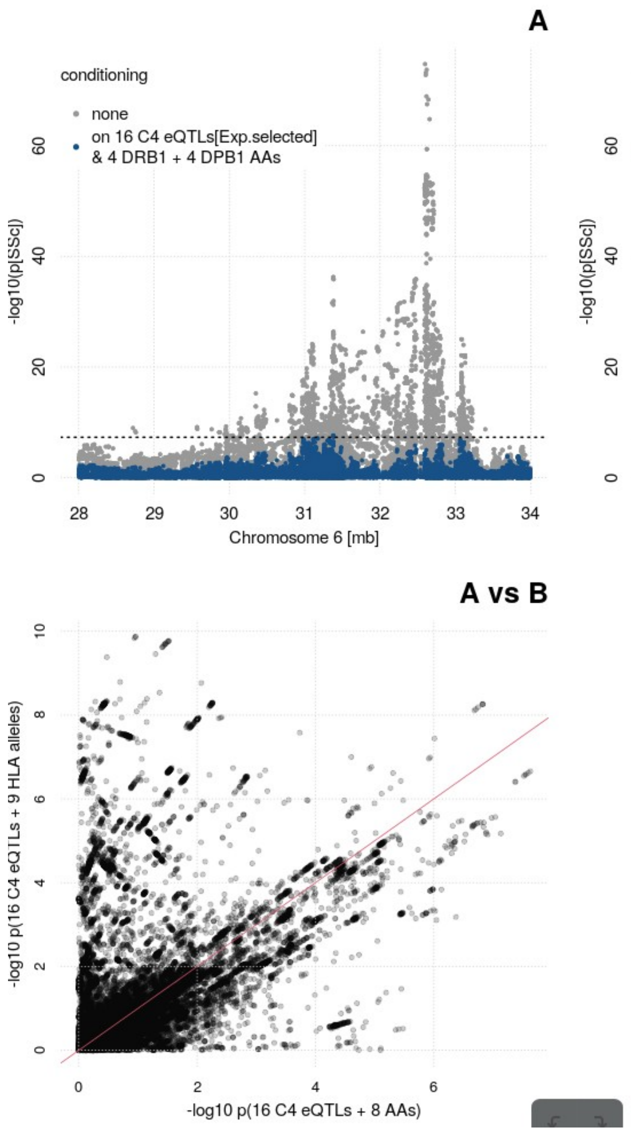

Supplementary Figure 5 MHC region conditional association with Systemic Sclerosis

Association is calculated using logistic regression in the first dataset (N=26,633) with cohort, genetic background and sex as covariates and depicted as position (GRCh38) by significance (Manhattan plot) in grey if no additional covariates were used. **A** Manhattan plot with additional conditioning on 16 independent *C4* eQTLs (obtained by forward selection in the second dataset) and 8 independent amino acids of DRB1 and DPB1 (obtained by forward selection in the first dataset conditioning on 16 independent eQTLs) depicted in blue. **B** Manhattan plot with additional conditioning on 16 independent *C4* eQTLs (obtained by forward selection in the first dataset) and 9 independent classical alleles of *DRB1* and *DPB1* (obtained by forward selection in the first dataset conditioning on 16 independent eQTLs) depicted in blue.

The second row depicts comparisons of the conditional p-values against each other.

Supplementary Table 1: Basic clinical epidemiology

* = percentages are estimated due to missing data. Cohort 1 has 9% missing data with respect to limited and diffuse SSc and 11% missing data on antibodies. Cohort 2 has 50% missing data with respect to limited and diffuse SSc and 25% missing data on antibodies.

	Cohort 1	Cohort 2
**total N**	26,633	857
**N, cases**	9,068	333
**N, controls**	17,565	524
**mean age, cases (sd)**	na	57.9 (sd: 13.5)
**mean age, controls (sd)**	na	46.9 (sd: 13.1)
**N, cases, female**	7,790	284
**N, cases, male**	1,278	49
**N, controls, female**	10,690	411
**N, controls, male**	6,875	113
**mean age, cases, female (sd)**	na	58.3 (sd: 13.4)
**mean age, cases, male (sd)**	na	55.5 (sd: 14.0)
**mean age, controls, female (sd)**	na	47.3 (sd: 13.1)
**mean age, controls, male (sd)**	na	45.3 (sd: 12.7)
**% limited cutaneous sclerosis ***	63	48
**% diffuse cutaneous sclerosis ***	28	34
**% anti-Centromer antibodies ***	42	47
**% anti-Topoisomerase antibodies ***	22	34

Supplementary Table 2A: Allele frequencies of 29 imputed C4 alleles across 10 countries

Allele names and codes are taken from Kamitaki Nature 2020

Allele	Code	Allele Frequency (AF)	(Kamitaki et al. 2020)	AF (Spain)	AF (France)	AF (Australia)	AF (Germany)	AF (Netherlands)	AF (USA)	AF (Italy)	AF (Sweden)	AF (UK)	AF (Norway)
BS	H_1_0_1_0	0.1101	0.137	0.0664	0.0896	0.1324	0.1139	0.1415	0.1098	0.0477	0.1366	0.1345	0.1233
BL	H_1_0_1_1	0.0029		0.0036	0.0026	0.0022	0.0018	0.0026	0.0038	0.0035	0.0022	0.0024	0.0014
AS	H_1_1_0_0	0.0005		0.0006	0.0005	0.0008	0.0001	0.0003	0.0003	0.0009	0.0003	0.0010	0.0000
AL	H_1_1_0_1	0.0563	0.048	0.1000	0.0696	0.0527	0.0454	0.0462	0.0510	0.0590	0.0474	0.0444	0.0571
BS-BL	H_2_0_2_1	0.0037		0.0029	0.0049	0.0029	0.0033	0.0041	0.0038	0.0038	0.0026	0.0045	0.0035
BL-BL	H_2_0_2_2	0.0017		0.0031	0.0038	0.0008	0.0018	0.0010	0.0013	0.0042	0.0001	0.0014	0.0001
AL-BS	H_2_1_1_1_A	0.0634	0.061	0.0516	0.0552	0.0670	0.0786	0.0533	0.0667	0.0706	0.0509	0.0640	0.0287
AL-BS	H_2_1_1_1_B	0.0479	0.045	0.0741	0.0527	0.0580	0.0309	0.0367	0.0441	0.0294	0.0320	0.0554	0.0278
AL-BS	H_2_1_1_1_C	0.0399	0.038	0.0655	0.0451	0.0323	0.0352	0.0362	0.0378	0.0554	0.0261	0.0317	0.0139
AL-BS	H_2_1_1_1_D	0.0408	0.045	0.0277	0.0498	0.0447	0.0419	0.0427	0.0413	0.0444	0.0365	0.0434	0.0296
AL-BS	H_2_1_1_1_E	0.0302		0.0237	0.0278	0.0384	0.0233	0.0273	0.0318	0.0185	0.0266	0.0374	0.0497
AL-BS	H_2_1_1_1_F	0.0109		0.0195	0.0088	0.0152	0.0036	0.0061	0.0113	0.0058	0.0049	0.0122	0.0025
AL-BS	H_2_1_1_1_G	0.0018		0.0040	0.0011	0.0007	0.0016	0.0003	0.0019	0.0042	0.0002	0.0010	0.0003
AL-BS	H_2_1_1_1_I	0.0071		0.0030	0.0037	0.0083	0.0109	0.0118	0.0061	0.0026	0.0127	0.0076	0.0082
AL-BS	H_2_1_1_1_X	0.0002		0.0003	0.0002	0.0001	0.0001	0.0001	0.0002	0.0006	0.0001	0.0003	0.0000
AL-BL	H_2_1_1_2_A	0.1384	0.155	0.1022	0.1147	0.1482	0.1548	0.1471	0.1478	0.0847	0.1642	0.1503	0.1615
AL-BL	H_2_1_1_2_B	0.2645	0.231	0.2752	0.2913	0.2131	0.2802	0.2567	0.2637	0.3977	0.2603	0.2230	0.3083
AL-BL	H_2_1_1_2_X	0.0030		0.0035	0.0033	0.0026	0.0045	0.0030	0.0036	0.0043	0.0015	0.0011	0.0008
AL-AS	H_2_2_0_1	0.0035		0.0045	0.0033	0.0040	0.0020	0.0022	0.0035	0.0033	0.0015	0.0045	0.0047
AL-AL	H_2_2_0_2_A	0.0383	0.032	0.0243	0.0313	0.0358	0.0445	0.0626	0.0368	0.0365	0.0492	0.0372	0.0433
AL-AL	H_2_2_0_2_B	0.0189	0.021	0.0194	0.0185	0.0165	0.0196	0.0179	0.0188	0.0204	0.0286	0.0175	0.0229
AL-AL	H_2_2_0_2_C	0.0391	0.045	0.0147	0.0335	0.0560	0.0265	0.0337	0.0438	0.0102	0.0415	0.0597	0.0502
AL-AL	H_2_2_0_2_X	0.0022		0.0019	0.0050	0.0021	0.0019	0.0015	0.0021	0.0021	0.0044	0.0021	0.0027
AL-BS-BS	H_3_1_2_1	0.0292		0.0590	0.0295	0.0238	0.0242	0.0121	0.0283	0.0457	0.0149	0.0210	0.0103
AL-BL-BS	H_3_1_2_2	0.0008		0.0010	0.0001	0.0008	0.0018	0.0009	0.0007	0.0012	0.0014	0.0003	0.0003
AL-BL-BL	H_3_1_2_3	0.0022		0.0045	0.0025	0.0009	0.0019	0.0018	0.0018	0.0047	0.0020	0.0013	0.0004
AL-AS-BS	H_3_2_1_1	0.0010		0.0007	0.0021	0.0005	0.0014	0.0012	0.0010	0.0005	0.0026	0.0006	0.0000
AL-AL-BS	H_3_2_1_2	0.0200		0.0155	0.0246	0.0199	0.0226	0.0278	0.0179	0.0181	0.0293	0.0182	0.0286
AL-AL-BL	H_3_2_1_3	0.0213		0.0277	0.0250	0.0193	0.0216	0.0213	0.0191	0.0200	0.0196	0.0223	0.0197

Supplementary Table 2B: Weighted imputation quality

Allele names and codes
are taken from Kamitaki Nature 2020

**C4**	0.90
**C4A**	0.91
**C4B**	0.90
**HERV-K**	0.90

**Allele**	**Code**	**R-square (imputation)**	**Allele Frequency (AF)**	**AF x r2 (C4)**	**AF x r2 (C4A)**	**AF x r2 (C4B)**	**AF x r2 (HERV)**
BS	H_1_0_1_0	0.95	0.1101	0.1046	0.1046		
BL	H_1_0_1_1	0.55	0.0029	0.0016	0.0016		0.0016
AS	H_1_1_0_0	0.59	0.0005	0.0003		0.0003	
AL	H_1_1_0_1	0.82	0.0563	0.0462		0.0462	0.0462
BS-BL	H_2_0_2_1	0.76	0.0037	0.0028	0.0028		0.0028
BL-BL	H_2_0_2_2	0.74	0.0017	0.0013	0.0013		0.0013
AL-BS	H_2_1_1_1_A	0.96	0.0634	0.0609	0.0609	0.0609	0.0609
AL-BS	H_2_1_1_1_B	0.96	0.0479	0.0460	0.0460	0.0460	0.0460
AL-BS	H_2_1_1_1_C	0.89	0.0399	0.0355	0.0355	0.0355	0.0355
AL-BS	H_2_1_1_1_D	0.93	0.0408	0.0380	0.0380	0.0380	0.0380
AL-BS	H_2_1_1_1_E	0.80	0.0302	0.0242	0.0242	0.0242	0.0242
AL-BS	H_2_1_1_1_F	0.94	0.0109	0.0103	0.0103	0.0103	0.0103
AL-BS	H_2_1_1_1_G	0.53	0.0018	0.0009	0.0009	0.0009	0.0009
AL-BS	H_2_1_1_1_I	0.67	0.0071	0.0047	0.0047	0.0047	0.0047
AL-BS	H_2_1_1_1_X	0.37	0.0002	0.0001	0.0001	0.0001	0.0001
AL-BL	H_2_1_1_2_A	0.94	0.1384	0.1301	0.1301	0.1301	0.1301
AL-BL	H_2_1_1_2_B	0.90	0.2645	0.2380	0.2380	0.2380	0.2380
AL-BL	H_2_1_1_2_X	0.82	0.0030	0.0025	0.0025	0.0025	0.0025
AL-AS	H_2_2_0_1	0.75	0.0035	0.0026		0.0026	0.0026
AL-AL	H_2_2_0_2_A	0.92	0.0383	0.0352		0.0352	0.0352
AL-AL	H_2_2_0_2_B	0.90	0.0189	0.0171		0.0171	0.0171
AL-AL	H_2_2_0_2_C	0.92	0.0391	0.0359		0.0359	0.0359
AL-AL	H_2_2_0_2_X	0.70	0.0022	0.0016		0.0016	0.0016
AL-BS-BS	H_3_1_2_1	0.91	0.0292	0.0266	0.0266	0.0266	0.0266
AL-BL-BS	H_3_1_2_2	0.73	0.0008	0.0006	0.0006	0.0006	0.0006
AL-BL-BL	H_3_1_2_3	0.66	0.0022	0.0014	0.0014	0.0014	0.0014
AL-AS-BS	H_3_2_1_1	0.65	0.0010	0.0006	0.0006	0.0006	0.0006
AL-AL-BS	H_3_2_1_2	0.86	0.0200	0.0172	0.0172	0.0172	0.0172
AL-AL-BL	H_3_2_1_3	0.72	0.0213	0.0154	0.0154	0.0154	0.0154

Supplementary Table 2C: Copy numbers of C4A, C4B, HERV-K, AS, AL, BS, BL per C4 allele

Copy numbers of the short (AS, BS) and long (AL, BL) form are inferred assuming 95% of C4A to contain HERV-K and thus be “long” (as reported in 4 independent publications, see methods). Allele names and codes are taken from Kamitaki Nature 2020

Allele	Code	copy numbers			copy numbers (probabilistic)			
		C4A	C4B	HERV-K	AS	AL	BS	BL
BS	H_1_0_1_0	0	1	0	0	0	1	0
BL	H_1_0_1_1	0	1	1	0	0	0	1
AS	H_1_1_0_0	1	0	0	1	0	0	0
AL	H_1_1_0_1	1	0	1	0	1	0	0
BS-BL	H_2_0_2_1	0	2	1	0	0	1	1
BL-BL	H_2_0_2_2	0	2	2	0	0	0	2
AL-BS	H_2_1_1_1_A	1	1	1	0.05	0.95	0.95	0.05
AL-BS	H_2_1_1_1_B	1	1	1	0.05	0.95	0.95	0.05
AL-BS	H_2_1_1_1_C	1	1	1	0.05	0.95	0.95	0.05
AL-BS	H_2_1_1_1_D	1	1	1	0.05	0.95	0.95	0.05
AL-BS	H_2_1_1_1_E	1	1	1	0.05	0.95	0.95	0.05
AL-BS	H_2_1_1_1_F	1	1	1	0.05	0.95	0.95	0.05
AL-BS	H_2_1_1_1_G	1	1	1	0.05	0.95	0.95	0.05
AL-BS	H_2_1_1_1_I	1	1	1	0.05	0.95	0.95	0.05
AL-BS	H_2_1_1_1_X	1	1	1	0.05	0.95	0.95	0.05
AL-BL	H_2_1_1_2_A	1	1	2	0	1	0	1
AL-BL	H_2_1_1_2_B	1	1	2	0	1	0	1
AL-BL	H_2_1_1_2_X	1	1	2	0	1	0	1
AL-AS	H_2_2_0_1	2	0	1	1	1	0	0
AL-AL	H_2_2_0_2_A	2	0	2	0	2	0	0
AL-AL	H_2_2_0_2_B	2	0	2	0	2	0	0
AL-AL	H_2_2_0_2_C	2	0	2	0	2	0	0
AL-AL	H_2_2_0_2_X	2	0	2	0	2	0	0
AL-BS-BS	H_3_1_2_1	1	2	1	0.05	0.95	1.9	0.1
AL-BL-BS	H_3_1_2_2	1	2	2	0.05	0.95	0.95	1.05
AL-BL-BL	H_3_1_2_3	1	2	3	0	1	0	2
AL-AS-BS	H_3_2_1_1	2	1	1	0.1	1.9	0.95	0.05
AL-AL-BS	H_3_2_1_2	2	1	2	0.05	1.95	0.95	0.05
AL-AL-BL	H_3_2_1_3	2	1	3	0	2	0	1

Supplementary Table 3: Beta values from the logistic regression analysis of the most complex model

The model contained cohort, sex and 5 principal components as co-variables next to the model terms. Logistic regression was calculated in the first dataset (N=26,633). Copy numbers (CN) were calculated from the imputed C4 alleles per individual as dosages.

Model Terms	beta	se	p
C4A CN	-0.09	0.11	4.2E-01
C4B CN	-0.04	0.10	6.9E-01
HERV-K CN	-0.07	0.10	5.1E-01
Sex:C4A	-0.17	0.06	3.4E-03
Sex:C4B	0.13	0.07	6.5E-02
HERV-K:C4A	0.04	0.02	3.2E-02
HERV-K:C4B	0.08	0.04	5.3E-02
C4A:C4B	-0.18	0.05	2.5E-04

Supplementary Table 4 A:
Evolution of models predicting the expression of total C4

Depicted are three models of rising complexity and their expression variance explained in the second dataset (N=857). Total C4 expression was obtained by summing C4A and C4B expression values. Copy numbers (CN) were calculated from the imputed C4 alleles per individual as dosages.

total C4 expression modeling:

Model	Predictors	beta	se	z-score	p.value	R-squared adjusted
1	totC4 CN	0.65	0.06	11.37	5.6E-28	0.13
2	totC4 CN	1.05	0.06	17.29	1.2E-57	0.27
	HERV-K CN	-0.57	0.04	-12.97	2.9E-35	
3	C4A CN	1.01	0.08	12.32	3.3E-32	0.27
	C4B CN	1.07	0.07	16.40	9.1E-53	
	HERV-K CN	-0.55	0.05	-10.93	4.2E-26	

Supplementary Table 4 B:
Evolution of models predicting the expression of C4A

Depicted are seven models and their expression variance explained in the second dataset (N=857). Copy numbers (CN) were calculated from the imputed C4 alleles per individual as dosages. Model 4 is the final model with only copy number information as predictors. Model 5 adds eQTLs by forward selection until no more eQTLs are found with p<0.01. Model 6 is an “eQTL only” model (forward selection until no more eQTLs are found with p<0.01). Model 7 is like model 6 but only eQTLs with suggestive association to SSc (pGWAS <10-5) are forward selected. All SNPs are GRCh38.

C4A expression modeling:

Model	Predictors	beta	se	z-score	p.value	R-squared adjusted
1	C4A CN	0.15	0.04	4.41	1.2E-05	0.02
2	C4A CN	0.22	0.05	4.89	1.2E-06	0.03
	HERV-K CN	-0.07	0.03	-2.33	2.0E-02	
3	AS CN	0.81	0.07	11.71	1.8E-29	0.14
	AL CN	0.10	0.03	2.89	4.0E-03	
4	AS CN	0.69	0.08	8.33	3.3E-16	0.21
	AL CN	0.26	0.05	5.35	1.1E-07	
	BS CN	0.34	0.07	4.68	3.3E-06	
	BL CN	0.35	0.04	8.75	1.1E-17	
5	AS CN	0.38	0.09	4.18	3.3E-05	0.40
	AL CN	0.24	0.04	5.33	1.3E-07	
	BS CN	0.40	0.07	5.55	3.9E-08	
	BL CN	0.20	0.05	4.16	3.5E-05	
	chr6.31646325.C.T	0.26	0.04	6.67	4.7E-11	
	chr6.32046798.A.G	0.09	0.03	2.54	1.1E-02	
	chr6.30985937.G.C	-0.65	0.15	-4.45	9.6E-06	
	chr6.32439525.A.G	-0.16	0.03	-4.70	3.0E-06	
	chr6.32049328.A.G	0.19	0.06	3.04	2.5E-03	
	chr6.31410581.T.G	0.12	0.03	3.66	2.7E-04	
	chr6.31493355.C.G	0.17	0.03	5.23	2.1E-07	
	chr6.32038878.C.T	-0.13	0.04	-3.43	6.3E-04	
	chr6.31858928.G.A	-0.27	0.06	-4.60	4.9E-06	
	chr6.32243441.A.G	-0.10	0.03	-3.40	7.1E-04	
	chr6.31955877.C.T	-0.14	0.04	-3.23	1.3E-03	
	chr6.32812693.C.T	-0.08	0.03	-2.89	4.0E-03	
6	chr6.31646325.C.T	0.70	0.19	3.73	2.1E-04	0.42
	chr6.32699394.A.T	-0.27	0.07	-3.92	9.4E-05	
	chr6.31953698.G.A	-0.61	0.04	-15.54	4.9E-48	
	chr6.31515359.A.G	0.16	0.03	5.68	1.8E-08	
	chr6.31955877.C.T	-0.39	0.04	-8.89	3.7E-18	
	chr6.31867387.G.C	-0.45	0.05	-8.57	5.1E-17	
	chr6.32288355.G.A	0.27	0.05	5.33	1.3E-07	
	chr6.31424341.A.G	0.08	0.03	2.93	3.5E-03	
	chr6.31964779.C.T	-0.58	0.09	-6.31	4.5E-10	
	chr6.32338313.G.A	-0.17	0.05	-3.39	7.3E-04	
	chr6.31720423.C.T	-0.23	0.05	-4.97	8.0E-07	
	chr6.30985937.G.C	-0.50	0.14	-3.56	4.0E-04	
	chr6.31869473.G.A	-0.21	0.04	-5.81	8.7E-09	
	chr6.32995105.G.A	-0.12	0.04	-3.07	2.2E-03	
	chr6.32635230.A.G	0.16	0.05	3.00	2.8E-03	
	chr6.31638745.C.G	-0.47	0.19	-2.54	1.1E-02	
	chr6.31359401.G.A	0.13	0.04	3.50	4.9E-04	
	chr6.31034675.A.G	-0.08	0.03	-2.92	3.6E-03	
	chr6.32700160.A.T	0.12	0.04	2.88	4.0E-03	
7	chr6.32370623.C.T	0.09	0.06	1.56	1.2E-01	0.33
	chr6.31741572.C.T	0.46	0.07	6.95	7.5E-12	
	chr6.32668221.G.T	-0.14	0.04	-3.12	1.9E-03	
	chr6.31720423.C.T	-0.38	0.06	-6.87	1.2E-11	
	chr6.31477568.T.C	-0.14	0.03	-5.17	3.0E-07	
	chr6.32094910.G.A	-0.23	0.03	-6.76	2.5E-11	
	chr6.32347100.A.G	-0.22	0.04	-6.06	2.0E-09	
	chr6.31711530.A.G	-0.32	0.06	-5.02	6.3E-07	
	chr6.31524676.G.A	-0.19	0.05	-4.25	2.4E-05	
	chr6.31435060.G.A	0.11	0.03	3.83	1.4E-04	
	chr6.31660956.C.T	-0.13	0.04	-3.49	5.1E-04	
	chr6.32418694.A.G	-0.21	0.05	-3.91	1.0E-04	
	chr6.32432311.C.T	0.12	0.04	2.91	3.7E-03	

Supplementary Table 4 C:
Evolution of models predicting the expression of C4B

Depicted are seven models and their expression variance explained in the second dataset (N=857). Copy numbers (CN) were calculated from the imputed C4 alleles per individual as dosages. Model 4 is the final model with only copy number information as predictors. Model 5 adds eQTLs by forward selection until no more eQTLs are found with p<0.01. Model 6 is an “eQTL only” model (forward selection until no more eQTLs are found with p<0.01). Model 7 is like model 6 but only eQTLs with suggestive association to SSc (pGWAS <10-5) are forward selected. All SNPs are GRCh38.

C4B expression modeling:

Model	Predictors	beta	se	z-score	p.value	R-squared adjusted
1	C4B CN	0.41	0.04	10.88	6.4E-26	0.12
2	C4B CN	0.38	0.04	10.51	2.3E-24	0.18
	HERV-K CN	-0.19	0.02	-7.79	1.9E-14	
3	BS CN	0.70	0.04	16.26	5.7E-52	0.24
	BL CN	0.17	0.04	4.12	4.2E-05	
4	AS CN	0.26	0.09	2.99	2.9E-03	0.27
	AL CN	0.22	0.05	4.44	1.0E-05	
	BS CN	0.82	0.08	10.50	2.4E-24	
	BL CN	0.22	0.04	5.29	1.5E-07	
5	AS CN	0.35	0.10	3.62	3.1E-04	0.39
	AL CN	0.20	0.05	4.03	6.1E-05	
	BS CN	0.56	0.09	6.36	3.2E-10	
	BL CN	0.06	0.05	1.23	2.2E-01	
	chr6.32082290.T.C	0.12	0.04	3.28	1.1E-03	
	chr6.31643381.T.C	0.16	0.04	3.71	2.2E-04	
	chr6.31301668.G.C	-0.13	0.04	-3.49	5.0E-04	
	chr6.32666452.C.T	0.13	0.04	3.74	2.0E-04	
	chr6.32715035.C.A	-0.14	0.06	-2.37	1.8E-02	
	chr6.31951801.A.G	0.16	0.04	4.06	5.3E-05	
	chr6.31413574.A.G	-0.10	0.03	-3.44	6.0E-04	
	chr6.32160617.C.T	-0.17	0.05	-3.61	3.3E-04	
	chr6.31865676.G.A	0.87	0.18	4.81	1.8E-06	
	chr6.31361056.G.A	-0.55	0.16	-3.43	6.4E-04	
	chr6.32242084.C.T	0.10	0.03	3.16	1.6E-03	
	chr6.32627454.T.G	0.21	0.05	4.01	6.6E-05	
	chr6.32632671.C.T	-0.16	0.05	-3.01	2.7E-03	
6	chr6.32375958.C.T	0.18	0.07	2.72	6.6E-03	0.38
	chr6.32160617.C.T	-0.27	0.05	-5.87	6.2E-09	
	chr6.31865676.G.A	1.28	0.18	7.30	6.8E-13	
	chr6.31508520.C.A	0.11	0.04	2.91	3.7E-03	
	chr6.31951801.A.G	0.18	0.03	5.25	1.9E-07	
	chr6.32245433.G.A	-0.15	0.03	-4.74	2.5E-06	
	chr6.32219945.C.T	-0.21	0.04	-5.05	5.4E-07	
	chr6.32102292.G.A	-0.35	0.04	-8.93	2.7E-18	
	chr6.31630746.C.T	-0.62	0.11	-5.44	6.8E-08	
	chr6.31425237.C.T	0.24	0.05	4.91	1.1E-06	
	chr6.31355959.T.A	-0.57	0.17	-3.43	6.4E-04	
	chr6.31646282.G.A	0.51	0.16	3.11	1.9E-03	
	chr6.31966200.A.C	0.17	0.05	3.16	1.6E-03	
	chr6.31361595.A.G	-0.10	0.04	-2.75	6.0E-03	
	chr6.32218370.C.A	-0.11	0.04	-2.66	8.0E-03	
7	chr6.31953698.G.A	-0.47	0.08	-6.05	2.2E-09	0.35
	chr6.31926849.A.G	0.25	0.09	2.87	4.2E-03	
	chr6.31515359.A.G	0.05	0.03	1.70	9.0E-02	
	chr6.31913532.T.C	0.37	0.05	6.76	2.5E-11	
	chr6.32347100.A.G	-0.15	0.03	-4.52	7.2E-06	
	chr6.31893364.A.T	-0.72	0.11	-6.24	7.0E-10	
	chr6.31890753.T.C	0.60	0.12	4.81	1.8E-06	
	chr6.31412627.C.T	0.20	0.05	3.78	1.7E-04	
	chr6.31335323.A.G	0.08	0.03	2.47	1.4E-02	
	chr6.31974114.T.G	1.55	0.34	4.62	4.4E-06	
	chr6.31879403.G.A	-1.28	0.33	-3.87	1.2E-04	
	chr6.31712097.G.T	0.37	0.09	4.09	4.7E-05	
	chr6.32215924.A.G	0.13	0.04	3.02	2.6E-03	
	chr6.32668221.G.T	-0.16	0.05	-3.39	7.4E-04	
	chr6.32371299.A.G	0.09	0.03	2.83	4.8E-03	

Supplementary Table 5: Evolution of models predicting blood serum concentrations of total C4

Depicted are six models of rising complexity and their C4 concentration variance explained in the second dataset (N=857). Copy numbers (CN) were calculated from the imputed C4 alleles per individual as dosages. Blood serum C4 concentrations have been normalized across 10+ laboratory sites to ensure data comparability
as described in Barturen Arthritis & Rheumatology 2021

blood serum C4 concentration modeling:

Model	Predictors	beta	se	z-score	p.value	R-squared adjusted
1	total C4 CN	0.05	0.01	7.22	1.3E-12	0.06
2	total C4 CN	0.07	0.01	9.06	9.9E-19	0.09
	HERV-K CN	-0.03	0.01	-5.42	7.9E-08	
3	total C4 CN	0.06	0.01	9.09	8.2E-19	0.11
	HERV-K CN	-0.03	0.01	-5.22	2.2E-07	
	Sex	0.04	0.01	3.61	3.3E-04	
	Age	0.00	0.00	2.46	1.4E-02	
4	total C4 CN	0.06	0.01	8.95	2.6E-18	0.12
	HERV-K CN	-0.03	0.01	-4.95	9.0E-07	
	Sex	0.04	0.01	3.52	4.6E-04	
	Age	0.00	0.00	3.24	1.2E-03	
	SSc	-0.02	0.01	-2.51	1.2E-02	
5	C4A CN	0.06	0.01	5.88	6.2E-09	0.12
	C4B CN	0.07	0.01	8.79	9.5E-18	
	HERV-K CN	-0.02	0.01	-3.73	2.0E-04	
	Sex	0.04	0.01	3.57	3.9E-04	
	Age	0.00	0.00	3.31	9.7E-04	
	SSc	-0.02	0.01	-2.61	9.4E-03	
6	AS CN	0.13	0.08	1.75	8.0E-02	0.12
	AL CN	0.03	0.01	4.42	1.1E-05	
	BS CN	0.06	0.01	7.81	1.9E-14	
	BL CN	0.05	0.01	5.32	1.4E-07	
	sex	0.04	0.01	3.58	3.6E-04	
	age	0.00	0.00	3.30	1.0E-03	
	SSc	-0.02	0.01	-2.58	9.9E-03	

Supplementary data 1: Pearson correlation of imputed allele dosages of HLA classical alleles and C4 alleles

Allele frequencies are noted for both types of alleles and correlations greater 0.4 are highlighted.
The column pGWAS(SSc) depicts the significance of assoctiation of HLA alleles to SSc as calculated by logistic regression in the first dataset (N=26,633) using 5 principal components, cohort and sex as covariates and $p<5x10^{-8}$ are depicted in bold. Allele names and codes are taken from Kamitaki Nature 2020

see Supplementary Data files

Supplementary data 2A: Residual associations of classical HLA alleles with SSc conditioning on 16 expression selected C4 eQTLs

Values are calculated by logistic regression in the first dataset (N=26,633)
using 5 genetic PCs, cohort, sex as covariates next to the C4 eQTLs.
$p<5 \times 10^{-8}$ are highlighted in bold.

see Supplementary Data files

Supplementary data 2B: Residual associations of HLA amino acids with SSc conditioning on 16 expression selected C4 eQTLs

Depicted are residual associations of HLA-amino acids to SSc using logistic regression conditioning on 5 PCs, cohort, sex and 16 expression selected C4 eQTLs

p values of genomewide significance are highlighted in bold

see Supplementary Data files
